# Explainable deep learning-based classification of Wolff-Parkinson-White electrocardiographic signals

**DOI:** 10.3389/fphys.2026.1855555

**Published:** 2026-09-23

**Authors:** Alice Ragonesi, Stefania Fresca, Karli Gillette, Stefan Kurath-Koller, Gernot Plank, Elena Zappon

**Affiliations:** 1 Department of Mechanical Engineering, University of Washington, Seattle, WA, United States; 2 Division of Biophysics and Medical Physics, Gottfried Schatz Research Center, Medical University of Graz, Graz, Austria; 3 Scientific Computing and Imaging Institute, University of Utah, Salt Lake City, UT, United States; 4 Department of Biomedical Engineering, University of Utah, Salt Lake City, UT, United States; 5 BioTechMed-Graz, Graz, Austria

**Keywords:** accessory pathway, deep learning, electrocardiogram, explainability, time series, Wolff-Parkinson-White

## Abstract

**Introduction:**

Wolff-Parkinson-White (WPW) syndrome is a cardiac electrophysiology (EP) disorder caused by the presence of an accessory pathway (AP) that bypasses the atrioventricular node, faster ventricular activation rate, and provides a substrate for atrio-ventricular reentrant tachycardia (AVRT). Accurate localization of the AP is critical for planning and guiding catheter ablation procedures. While traditional diagnostic tree (DT) methods and more recent machine learning (ML) approaches have been proposed to predict AP location from surface electrocardiogram (ECG), they are often constrained by limited anatomical localization resolution, poor interpretability, and the use of small clinical datasets.

**Methods:**

In this preliminary patient-specific study, we present a Deep Learning (DL) model for the localization of single manifest APs across 24 cardiac regions, trained on a large, physiologically realistic database of synthetic ECGs generated using a personalized virtual heart model. We also integrate eXplainable Artificial Intelligence (XAI) methods, Guided Backpropagation, Grad-CAM, and Guided Grad-CAM, into the pipeline. This enables interpretation of DL decision-making and addresses one of the main barriers to clinical adoption: lack of transparency in ML predictions.

**Results:**

On the considered single heart geometry, our model achieves a localization accuracy above 94% and an average F1 score over 93%. XAI outputs are physiologically validated against known depolarization patterns, and a novel index is introduced to identify the most informative ECG leads for AP localization.

**Discussion:**

Results highlight lead V2 as the most critical, followed by aVR, and aVL in the left ventricle (LV) and V1 in the right ventricle (RV). This work demonstrates the potential of combining cardiac digital twins with explainable DL to enable accurate, transparent, and noninvasive AP localization.

## 1 Introduction

WPW syndrome is a heart rhythm disorder characterized by the presence of at least one AP between the atria and the ventricles that bypasses the atrioventricular node (Vătăsescu et al., 2024). These APs enable conduction outside the normal nodal pathway, which can facilitate abnormally fast ventricular rates, as atrial arrhythmias are transmitted directly to the ventricles without the filtering effect of the atrioventricular node. When antegradely conducting, APs cause ventricular pre-excitation (Sapra et al., 2020), altering the normal sequence of ventricular activation. Furthermore, the presence of an AP provides a substrate for atrioventricular reentrant tachycardia, which may occur through orthodromic or antidromic conduction circuits, producing narrow or wide QRS complexes, respectively. WPW affects up to 0.5% of the general population (Pærregaard et al., 2023; Elendu et al., 2025), with possible symptoms such as paroxysmal supraventricular tachycardia, atrial fibrillation, atrial flutter, ventricular fibrillation, and, eventually, sudden death depending on the manifestation and location of the pathways (Pappone et al., 2012; Sapra et al., 2020; Schiavone et al., 2024). Radiofrequency catheter ablation is the first-line therapy, aiming to eliminate the AP, re-establish a non-pre-excited sinus rhythm, and impede the formation of atrioventricular re-entry tachycardias (AVRTs). However, approximately 6% of ablation procedures remain unsuccessful (Fujino et al., 2020; Sherdia et al., 2023).

Accurate localization of the AP prior to ablation is crucial for optimal procedural planning, reducing time for intervention and associated risks. Traditionally, single manifest AP localization relies on analysis of the surface ECG, either acquired during resting sinus rhythm (Lindsay et al., 1987; Milstein et al., 1987; Fitzpatrick et al., 1994; Xie et al., 1994; Chiang et al., 1995; d’Avila et al., 1995; Iturralde et al., 1996; Arruda et al., 1998; Boersma et al., 2002; Kurath-Koller et al., 2022; El Hamriti et al., 2023; Khalaph et al., 2025) or under atrial pacing (Fananapazir et al., 1990; Pambrun et al., 2018; Kurath-Koller et al., 2022). Characteristic ECG features in WPW syndrome with antegrade APs include a short PR interval, the presence of a delta wave, and a wide QRS complex. Localization algorithms, typically structured as diagnostic decision trees, associate these morphological features in selected leads with predefined anatomical regions along the mitral valve (MV) or tricuspid valve (TV) annulus. For instance, Arruda et al. (1998) proposed a diagnostic algorithm based on the polarity of the delta wave in leads I and V1 to determine whether the AP is located along the MV or TV. To further refine localization, anteroseptal, posteroseptal, or lateral, they assessed the positivity, negativity, or isoelectricity of leads II, aVF, V1, and I. In contrast, more recent algorithms, such as those in Pambrun et al. (2018), El Hamriti et al. (2023), and Khalaph et al. (2025), relied on the overall QRS complex polarity in lead V1 to determine left versus right annular location. These latter algorithms then used the polarity of the full QRS complex, rather than the delta wave alone, in inferior leads (II, III, aVF) and sometimes counted the number of positive and negative deflections to classify the AP as anterolateral, posterolateral, or septal. However, a study employing a computational model for simulating WPW syndrome with single manifest antegrade AP, Gillette et al. (2025) showed that the largest variations in QRS morphology and delta wave due to the presence of an AP were found in lead V2 for both LV and RV, while a very similar QRS complex was obtained in lead V1, especially for antegrade AP located in the septum. Furthermore, manual classification of AP location based on DTs can be complex, time-consuming, and highly prone to inter-observer variability in identifying ECG patterns (McGavigan et al., 2007; Teixeira et al., 2016), leading to an overall low predictive accuracy. Consequently, the development of rapid, automated, objective and non-invasive approaches to analyze surface ECG signals is of growing interest to improve the identification of APs before ablation.

ML techniques have previously been applied to classify various cardiac pathologies based on morphological features extracted from the ECGs (Jones et al., 2020; van de Leur et al., 2021; Jahmunah et al., 2022; Le et al., 2022; van de Leur et al., 2024a). Unlike traditional approaches that rely on manually derived features, modern ML methods, particularly DL, enable automated extraction and identification of patterns within complex signals (van de Leur et al., 2024b). This automated analysis improves efficiency, reduces subjectivity, and significantly decreases the time and effort required for interpretation (Nishimori et al., 2021; Senoner et al., 2021; de Oliveira Bernardo, 2023; van de Leur et al., 2024b; Yahyazadeh et al., 2024; Hennecken et al., 2025). ML-based algorithms for the classification of single manifest APs had been proposed in a number of works. For example, Yahyazadeh et al. (2024) implemented an automatic feature extraction based on the McSharry model to derive quantitative parameters from ECG traces of 31 patients with WPW, focusing on the polarity of the delta wave and the morphology of QRS. A random forest and a gradient boosting approach were then employed to assign the obtained features to either right or left ventricles AP location, achieving a better accuracy compared to traditional DTs. A combination of manual and automated methods for extracting ECG features, such as delta wave polarity and QRS morphology, was employed by Bernardo et al (de Oliveira Bernardo, 2023). These features were subsequently fed to random forest and gradient boosting algorithms to classify the AP location in 120 patients as left, septal, or right. DL techniques have been deployed in more recent studies (Nishimori et al., 2021; Senoner et al., 2021; Hennecken et al., 2025). Senoner et al. (2021) utilized a neural network with back propagation to classify APs in 107 patients across 14 distinct anatomical locations. Nishimori et al. (2021) developed a hybrid model combining ECG signals with a Convolutional Neural Network (CNN)-based encoding of chest X-ray images, feeding these into a CNN to classify 206 APs into three anatomical groups. More recently, Hennecken et al. (2025) applied a causal neural network with a multi-task learning framework to localize APs in 645 patients, categorizing them as right-sided, septal, or left-sided.

Independent of the deployed technique, either ML methods or traditional DTs generally demonstrate acceptable accuracy in identifying lateral AP positions along the TV and MV annuli. However, their accuracy in predicting septal APs remains limited. Most existing DTs are designed to determine the circumferential (rotational) location of the AP, i.e., assigning it to broad regions encircling the MV or TV. These models, however, fail to consider the possible longitudinal (apex-to-base) position of the AP, which would allow for more precise localization of the AP along the LV and RV. As a result, current algorithms rely on a coarse subdivision into a limited number of regions defined circumferentially around the valve annuli, substantially restricting their ability to pinpoint the AP site, especially along the apico-basal direction.

ML-based techniques have generally demonstrated superior accuracy compared to traditional DT approaches for localizing accessory pathways. However, the performance of these models is often constrained by the limited size of the training datasets, which significantly affects both the robustness and generalizability of the results. The availability of large, high-quality annotated datasets is critical for the effective training and validation of ML models. However, acquiring such datasets, particularly in the context of rare or heterogeneous EP conditions, remains a key limitation, hindering the full potential of data-driven approaches in this domain. Furthermore, ML techniques are considered black-box models, since their internal decision-making processes lack transparency, and can thus undermine clinical trust and diminish their acceptance in medical practice (Ayano et al., 2022; Siontis et al., 2023).

To address the limitations posed by small clinical datasets, virtual models of cardiac EP that accurately replicate activation and repolarization in patients offer a compelling solution. These models enable the simulation of all cardiac activation patterns relevant in WPW patients, including anti- and orthodromic AVRTs, such as those observed in WPW syndrome, in a controlled, reproducible, and scalable manner (Corral-Acero et al., 2020; Loewe et al., 2022). When personalized in terms of both cardiac anatomy and EP properties, often referred to as cardiac digital twins, these models can be leveraged for diagnostic refinement, prognostic evaluation, and treatment planning (Bhagirath et al., 2024; Dasí et al., 2024; Qian et al., 2025). Furthermore, they provide a valuable platform for generating synthetic data to supplement limited clinical datasets, enhancing the training and reliability of ML algorithms (Doste et al., 2022; Gillette et al., 2023). In this context, Gillette et al. (2025) developed a subject-specific computational model of WPW syndrome to investigate how variations in AP location influence ECG morphology, by creating a larger synthetic cohort of ECGs compared to the ones previously used in AP localization works.

To mitigate concerns over transparency and reliability, XAI methods (Samek and Müller, 2019) have been developed and applied in ECG classification tasks, helping to clarify how predictions are made. However, to the best of our knowledge, no study has so far implemented these techniques in the context of WPW syndrome. Incorporating XAI into ML models for AP localization would greatly enhance transparency, physician confidence, and clinical applicability (Jones et al., 2020; van de Leur et al., 2021; Jahmunah et al., 2022; Le et al., 2022; van de Leur et al., 2024a).

Building on the virtual database of antegrade single manifest APs introduced in (Gillette et al., 2025), this work presents a DL-based model for the localization of APs across 24 distinct cardiac regions. The model is integrated with XAI techniques to enhance transparency and explainability of the DL inference process. Using a calibrated digital twin of a single subject in sinus rhythm, the simulated APs produce significant variation in ECG morphology, spanning a broad and physiologically relevant range of possible AP locations. This results in a rich and comprehensive dataset of 8842 12-lead ECGs suitable for learning the relationship between AP position and surface ECG features. The 24 selected regions account for both rotational and longitudinal positioning of the AP, thereby enabling more precise localization. To explore optimal input data dimensionality to maximize the network feature extraction capability, we tested three different CNN architectures. To select the best XAI method to define model transparency, we implemented and compared three XAI techniques for ECG classification interpretation in the form of Guided Backpropagation (Springenberg et al., 2015), Grad-CAM, and Guided Grad-CAM (Selvaraju et al., 2020). Although initially developed for image data, these methods have recently demonstrated strong potential in identifying ECG morphological features associated with specific cardiac pathologies (Jones et al., 2020; van de Leur et al., 2021; Jahmunah et al., 2022; Le et al., 2022; van de Leur et al., 2024a).

The evaluation of our novel DL-based method on a high-fidelity synthetic dataset with known ground truth suggests a potential improvement over traditional DTs and existing ML approaches. In this setting, the model achieved a localization accuracy and an F1 score above, respectively, 94% and 93% across the 24 regions. However, these findings are based on data from a single subject and should therefore be interpreted as preliminary. Furthermore, we clustered similar XAI outputs within each region and explored their relationship with EP observations of depolarization patterns caused by APs, providing an initial indication of physiological consistency in the model decision-making process. Finally, we introduce a novel index to summarize XAI results and identify potentially informative leads for AP detection in the left and right ventricles. While our observations are in line with those reported in (Gillette et al., 2025), notably the prominent role of lead V2, followed by aVL and V1, with leads such as I and II, typically relevant in DTs, appearing less informative, these conclusions require further validation on larger and more diverse datasets, including real-world recordings. Overall, the results provide encouraging early evidence, but more comprehensive analysis is needed to confirm the robustness and generalizability of the proposed method.

The paper is organized as follows: Section 2 presents the overall methodology, including the generation of the dataset (Section 2.1), the CNN architectures (Section 2.2), and the XAI techniques (Section 2.4). Section 3 reports the results, including the accuracy of the proposed methodology (Section 3.1), the physiological interpretation of the XAI outputs (Section 3.2), and the proposed index for summarizing XAI relevance (Section 3.3). The discussion, limitations, and conclusions are provided in Sections 4, 5, and 6, respectively.

## 2 Methods

In this section, we describe the DL models employed for the ECG classification, together with the training and testing datasets. Furthermore, the explored XAI methods for the interpretation of the DL decision-making process are discussed. Overall, three Fully Convolutional Networks (FCNs) were trained, validated, and tested on synthetic ECG data. For interpretability, only the network with 2D kernels was retained, as it allowed the computation of lead-specific feature relevance using Guided Grad-CAM saliency maps. Explainability analysis was performed exclusively on correctly classified test samples. XAI results were evaluated both globally – across all samples, to define a lead importance index – and locally – through saliency maps of representative ECGs – to assess the alignment between model attention and physiologically meaningful features. The implemented code will be made available in a Git repository.

### 2.1 Dataset

The dataset used in this work consists of the *in silico* 12-lead pathological ECGs dataset generated in (Gillette et al., 2025), and publicly available on the Zenodo platform (doi: 10.5281/zenodo.10949803). A cardiac digital twin of one healthy subject was created, comprising a four-chamber heart and the corresponding torso, and calibrated using the subject’s 12-lead ECG. A set of pre-ablation APs was subsequently generated based on physiological constraints, including physiological distance from the atrial valves. Each AP entry site in the ventricular myocardium was expressed through the rotational (*ϕ*), apicobasal (*z*) and transmural (*ρ*) universal ventricular coordinates (UVCs). The APs, along with the corresponding ECGs, were grouped into 12 regions per ventricle, similar to the AHA model, resulting in *C* = 24 regions serving as the ECGs labels for the classification task (see **Figure 1**). The rotational and apico-basal UVCs were used as reference cutoffs to delineate the anatomical regions for AP localization. The rotational coordinate sliced the ventricles around the mitral and tricuspid valve, while the apico-basal coordinate bisected each slice. The septal APs were attributed to regions in the LV. In total, the dataset comprised *N* = 8842 ECGs, with 4506 associated to APs in the LV and 4338 to APs in the RV. The ECG time series was restricted to the [100,300] ms interval, comprising *T* = 200 time steps, corresponding to the portion of the QRS complex. Indeed, morphological features in the QRS complex have been physiologically linked to WPW syndrome (Fananapazir et al., 1990; Haissaguerre et al., 1994). During this interval, R peaks were not aligned, as the timing of pre-excitation, while shifting signals along the time axis, could potentially be a characteristic upon which a classifier learns to assign samples to regions. Anatomical regions and data variability associated to each region can be seen in **Figures 1**–**3**.

**Figure 1:**
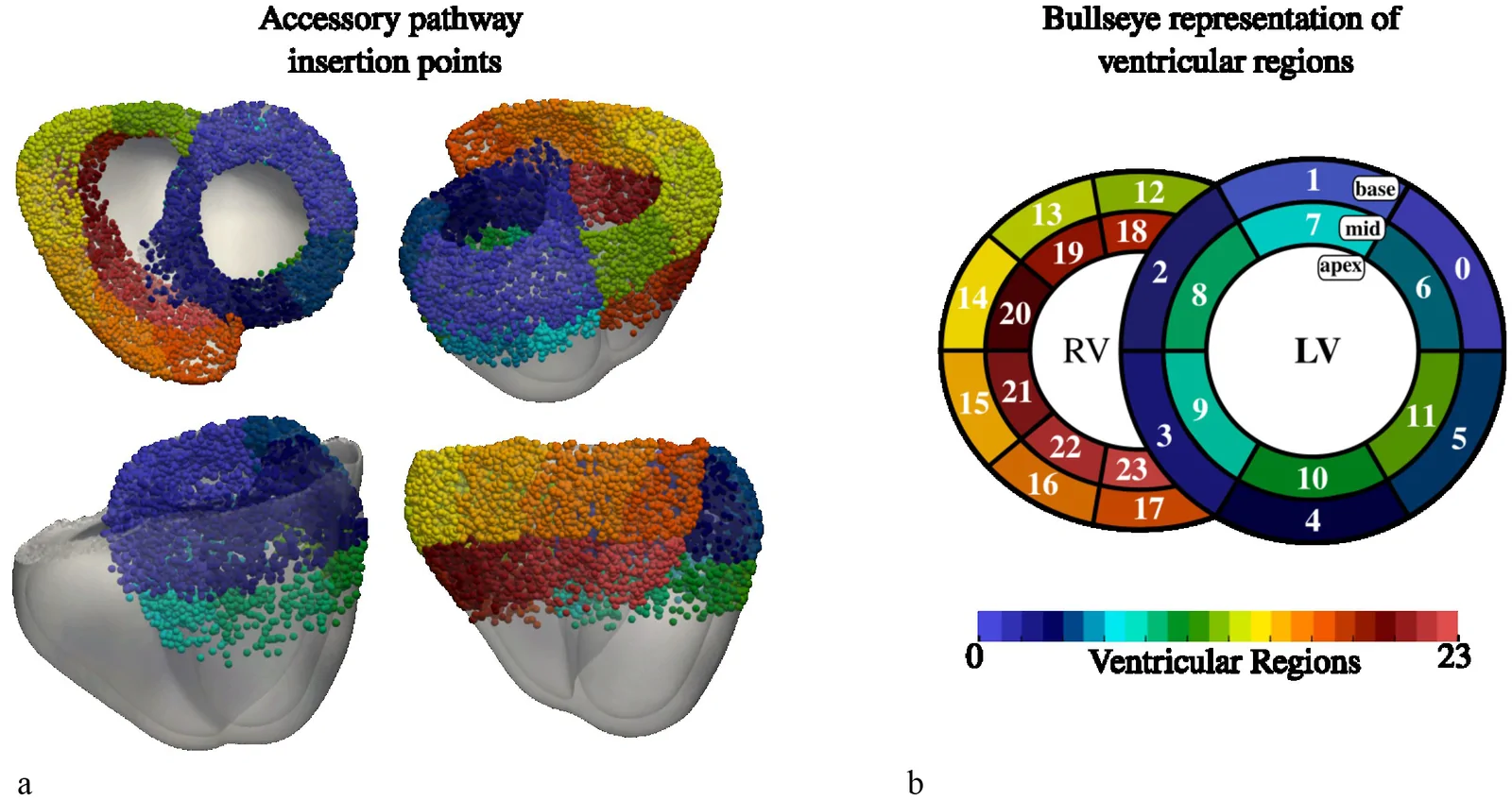
AP insertion points grouped according to the defined regions **(a)** and bullseye representation of the anatomical region distribution **(b)**. The regions were defined based on UVC, including both rotational and longitudinal subdivision.

**Figure 2:**
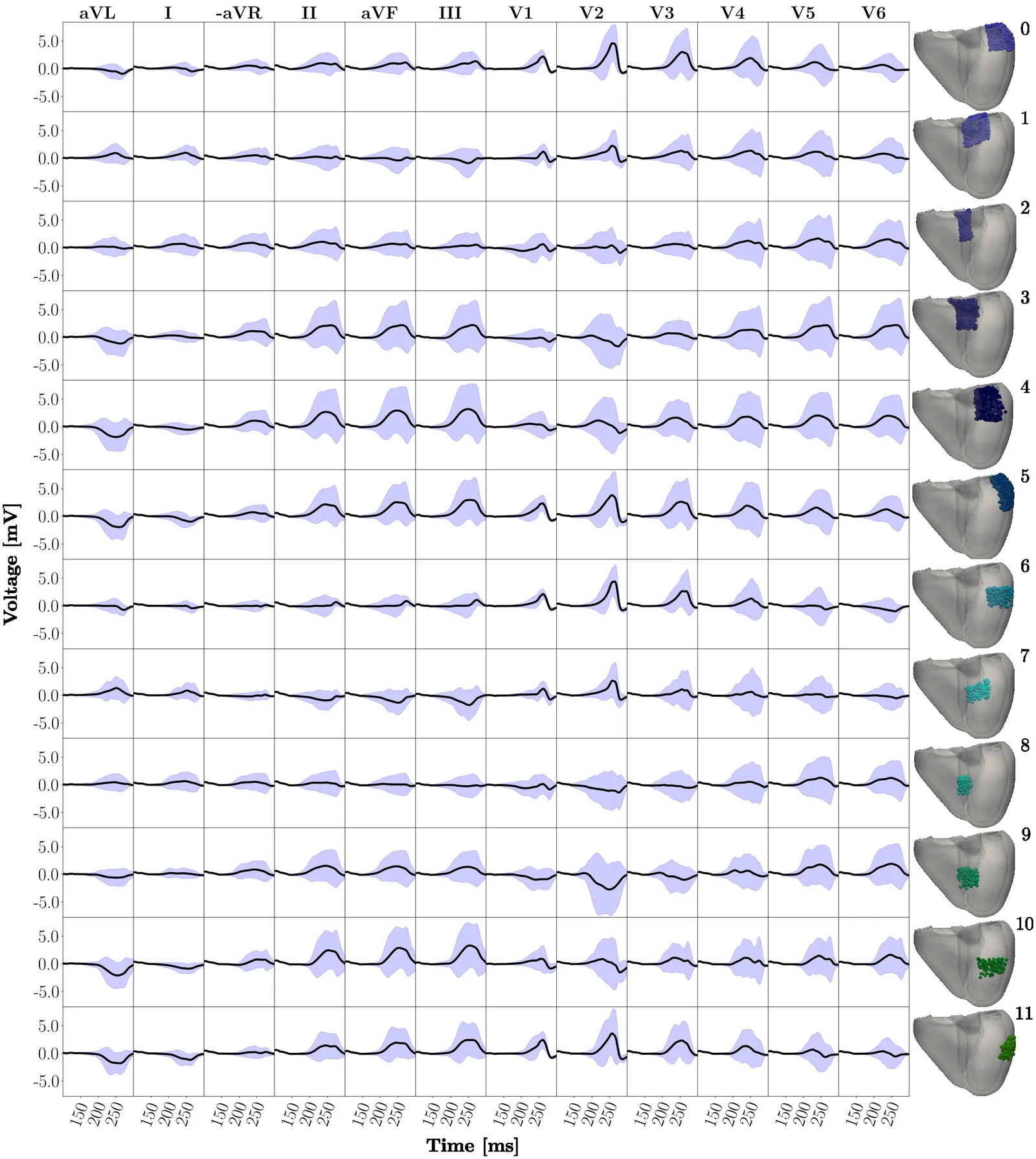
Regional variation of the signals with AP in the LV. Mean signal (black) with shaded regions representing plus or minus 2 standard deviations across samples. Next to the leads, the APs insertion sites in the ventricles are displayed together with the region numbering.

**Figure 3:**
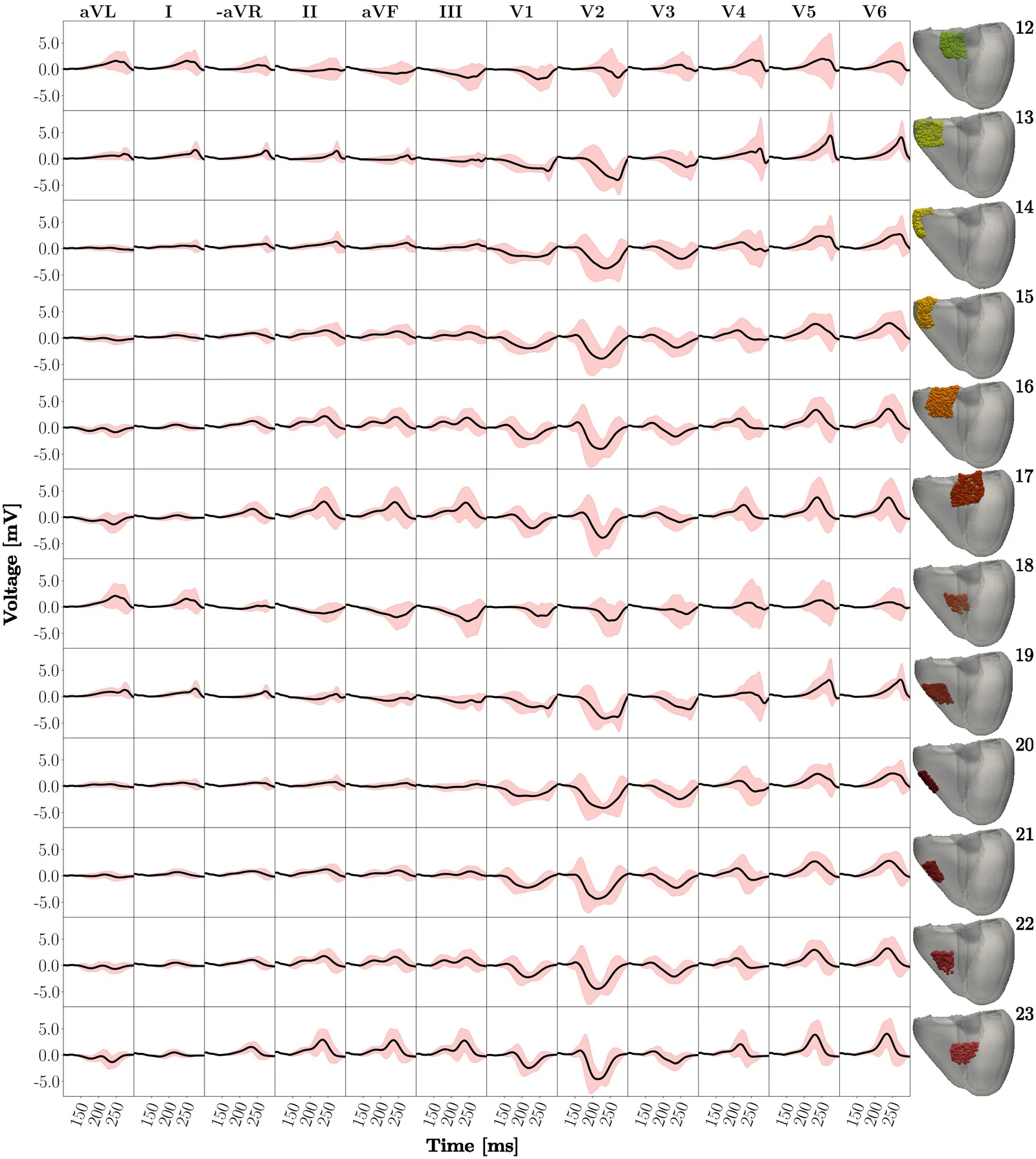
Regional variation of the signals with AP in the RV. Mean signal (black) with shaded regions representing plus or minus 2 standard deviations across samples. Next to the leads, the APs insertion sites in the ventricles are displayed together with the region numbering.

From a mathematical standpoint, 12-lead ECGs are multivariate time series with *L* = 12 channels, or dimensions, each representing the voltage in a lead over time. Each instance can be easily treated as a matrix $S=\left[s_{1};\ldots ;{\text{s}}_{L}\right]\in {\mathbb{R}}^{T\times L}$, where $s_{i}\in {\mathbb{R}}^{T}$, *i* = 1,…, *L*, stores the univariate time series associated to a single lead. Other representations of the ecg signals are possible by reshaping **S**, and were explored in this paper to learn multiple classifiers. Thus, in the following, **S***_r_* will refer to the reshaped version of matrix **S**.

### 2.2 Architectures

Multiple nonlinear FCN (Fawaz et al., 2019) classifiers $G\left(S_{\text{r}};\text{Ω}\right):{\mathbb{R}}^{D}\to {\left[0,1\right]}^{C}$ were trained to predict the probabilities $\hat{y}={\left({\hat{y}}_{0},\ldots ,{\hat{y}}_{C-1}\right)}^{T}\in {\mathbb{R}}^{C}$ of a signal **S***_r_*, formatted as to have dimensionality *D*, of belonging to each class *c* = 0,…, *C* − 1, based on an optimal set of weights Ω. The core structure common to all fcn models consisted of ℒ=3 consecutive convolutional blocks, each comprising a convolutional layer followed by a BatchNormalization for regularization (Ioffe and Szegedy, 2015) and a ReLU activation (Glorot et al., 2011). The final block preceded a Global Average Pooling (GAP) layer (Lin et al., 2014), whose output was passed through a fully connected layer and a Softmax activation (Bishop, 2006). The convolutions were used to make the classes linearly separable and to filter the most important features of the signal **S***_r_* (Haykin, 2009). In the *i*-th convolutional block, i=1,…,ℒ, *M_i_* convolutional filters are swept over the input **X***_i_*_−1_, with stride *s_i_* controlling the step size at which the filters move across the input. After the BatchNormalization ℬN and ReLU transformations, a multivariate feature map $X_{i}=\left[x_{i}^{1};\ldots ;x_{i}^{{\text{M}}_{\text{i}}}\right]\in {\mathbb{R}}^{D_{i}\times M_{i}}$ with *M_i_* channels is provided as output, such that each channel *m* = 1,…, *M_i_* holds the following nonlinear representation of the input:

(1) $$x_{i}^{m}=\text{ReLU}\left(ℬN\left(\sum_{j=1}^{M_{i-1}}w_{i,j}^{m}{*}_{s_{i}}x_{i-1}^{j}\right)\right),$$

where ${*}_{s_{i}}$ denotes the convolution operation with stride *s_i_*, $w_{i,j}^{m}$ is the set of weights defining the *j*-th kernel of filter *m* in the *i*-th convolutional layer, and $X_{0}=S_{r}$. The dimensionality *D_i_* of the features obtained for each channel is affected by the input’s shape, as discussed in the following in Sections 2.2.1-2.2.3.

After the nonlinear transformations, the GAP layer is used to flatten the multivariate feature map $X_{ℒ}$ into the vector $v\in {\mathbb{R}}^{M_{ℒ}}$ collecting the average of each channel, then linearly mapped by the fully connected layer to the vector of logits $z=\left(z_{0},\ldots ,z_{C-1}\right)\in {\mathbb{R}}^{C}$. Finally, the Softmax activation function is employed to perform the classification, that is, to compute the probabilities ${\hat{y}}_{c}$, *c* = 0,…, *C* − 1, such that


$${\hat{y}}_{c}=\frac{e^{z_{c}}}{\sum_{j=0}^{C-1}e^{z_{j}}}.$$

The training was performed on the training set *I_train_*, employing a gradient descent algorithm to minimize the cross-entropy loss function


$$CCE\left(y,\hat{y}\right)=-\sum_{c=0}^{C-1}y_{c}\log\left({\hat{y}}_{c}\right).$$

between the ground truth **y** and predicted $\hat{y}$, where **y** is a one-hot-encoded label vector $y=\left(y_{0},\ldots ,y_{C-1}\right)\in {\mathbb{R}}^{C},{\text{y}}_{c}\in {\left\{0,1\right\}}^{C}$

In this work, the core FCN architecture was kept unchanged while evaluating its classification performance under different input configurations, leading to three distinct FCN models. Specifically, the ECG was represented as: (i) a univariate time series formed by sequentially stacking all leads, resulting in a series of length *L · T* (Section 2.2.1); (ii) a multi-dimensional signal comprising *L* separate one-dimensional time series (Section 2.2.2); and (iii) a two-dimensional image with height *T* and width *L* (Section 2.2.3). Indeed, variations in input dimensionality called for differences in the neural network architectures, in terms of the number of channels at the input layer and kernel dimensionality.

#### 2.2.1 Single-channel FCN with 1D kernels

One strategy for processing multivariate time series is to reshape each data sample **S** into a longer univariate sequence $S_{r}={\left[s_{1}^{T};\ldots ;s_{L}^{T}\right]}^{T}\in {\mathbb{R}}^{T\cdot L}$ by stacking the individual dimensions end-to-end. In such a setting, the convolutional layers are constituted of 1D convolutional kernels sliding over the flattened sequence. The kernel length must therefore be carefully set to capture dependencies both within individual dimensions (intra-dimensional) and across different dimensions (inter-dimensional).

In this configuration, the 12 leads were stacked to represent each ECG as a univariate sequence of 2400 time steps, which was then processed by a FCN with a single input channel. For each convolutional layer, the number of kernels, kernel size, and stride were treated as tunable hyperparameters. Given the absence of prior knowledge regarding whether the most informative features for classification resided within individual leads or across inter-lead relationships, the design prioritized the extraction of pathology-relevant features at the lead scale. It was hypothesized that employing kernel sizes comparable to the full length of the stacked leads could result in excessive information loss. Accordingly, kernel sizes approximately matching the length of a single lead were preferred, with values up to 100 permitted in the first convolutional layer. The stride parameter

was also tuned, as maintaining a fixed stride of 1 – as implemented in Sections 2.2.2 and 2.2.3 – could result in the extraction of redundant features due to the effects of the convolution and weight sharing (see Equation 1), particularly when used in conjunction with larger kernels. Accordingly to input shape and network hyperparameters, the feature maps extracted by the convolutional layers were multivariate time series $X_{i}\in {\mathbb{R}}^{T_{i}\times M_{i}}$, with length $T_{i}<T\cdot L$, i=1,…,ℒ, as a consequence of the stride being larger than one.

#### 2.2.2 Multi-channel FCN with 1D kernels

As an alternative input formatting, each ECG signal was arranged into a matrix, i.e. $S_{r}=S\in {\mathbb{R}}^{T\times L}$, which was fed into a FCN with *L* input channels – one for each dimension of the multivariate time series, as in Rosafalco et al. (2021). In this configuration, a single 1D signal is fed to each input channel of the FCN, thus requiring 1D kernels for performing the convolutions, with kernel lengths to be optimized through tuning operations. Here, 1D kernels with appropriate padding and a stride of 1 extract one dimensional feature maps, i.e. time series, with length equal to the input. Therefore, considering *M_i_*channels, the generated latent representations are *T* steps long multivariate time series $X_{i}\in {\mathbb{R}}^{T\times M_{i}}$.

Consequently to the formatting, the features extracted channel wise were combined through Equation 1 already at the first layer, thus allowing the network to optimize a summary representation of the features gathered from all leads.

#### 2.2.3 Single-channel FCN with 2D kernels

In the final approach to ECG classification, the signals were treated as single-channel images, similarly to the method proposed by Ramírez et al (Ramirez et al., 2024). Each signal was represented as a tensor $S_{r}\in R^{T\times L\times 1}$, where the height and width of the image corresponded to the signal length *T* and the number of leads *L*, respectively. This representation necessitated the use of two-dimensional convolutions, wherein squared 2D kernels were employed to capture both inter-lead and intra-lead relationships, learning to represent them in 2D feature maps, i.e. $X_{i}\in {\mathbb{R}}^{T\times L\times M_{i}}$, using circular padding along the lead axis: for a kernel of size *k*, each convolution borrows the (*k −*1)*/*2 leads from the opposite edge of the image (matching the padding width a same-size zero-padded convolution would use), so every lead is convolved with an equally populated, real-valued neighborhood regardless of its position in the image. The rationale for using non-zero padding is detailed in the **Supplementary Materials**, alongside the analysis of XAI-method robustness under lead permutations.

Treating ECGs as images implied a one-to-one correspondence between image columns and ECG leads, which was subsequently leveraged to interpret the individual contributions of different leads to the classification outcomes, as discussed in Section 2.4.

### 2.3 Comparison with established algorithms

The performance of our best model was evaluated against the DT EASY-WPW algorithm proposed by El Hamriti and coauthors (El Hamriti et al., 2023), as well as the DT algorithm developed by Arruda and coauthors (Arruda et al., 1998). To account for differences in anatomical subdivisions across reference regions, the ECGs in our dataset were regrouped according to the DT region definitions in (El Hamriti et al., 2023) and (Arruda et al., 1998). The complete description of the new ground truth subdivision and of the overall comparison procedure is given in detail in the **Supplementary Material**. The regrouping subdivision was reviewed and approved by a cardiologist with expertise in WPW syndrome, who is a co-author of this study. For comparison, DTbased localization was also performed by the WPW expert cardiologist blinded to the ground-truth AP regions.

It should be noted that this comparison is based on a limited and synthetic dataset derived from a single subject. Therefore, the results should be interpreted as an initial, exploratory assessment rather than a definitive benchmark. To compare classification performance, a one-sided McNemar’s exact test was applied to the contingency table of correctly versus incorrectly classified samples, using a significance threshold of *α* = 0.05.

### 2.4 Network explainability

Interpreting deep learning models, especially CNNs, remains a significant challenge due to their inherent “black-box” nature. Gaining insight into the features these models learn is essential for enhancing transparency and building trust in their predictions. In this work, we investigate the use of *post-hoc* XAI techniques – namely, Grad-CAM (Selvaraju et al., 2020), Guided Backpropagation (Springenberg et al., 2015), and their combination, Guided Grad-CAM (Selvaraju et al., 2020). The results were tested under standard XAI sanity checks and independently reviewed by a cardiologist with expertise in WPW syndrome, who is a co-author of this study. The aforementioned analyses are reported in the **Supplementary Materials**.

*Post-hoc* XAI methods operate by attributing a relevance score to input features, effectively defining a scalar function that ranks, by ascending score values, the importance of each feature with respect to the classifier output (Theissler et al., 2022). The resulting set of scores, termed a saliency map, explains the influence of input components on the computation of a specific model prediction, typically a logit *z_c_*of the last fully connected layer, associated with class *c*.

Among the three investigated techniques, Guided Backpropagation produces high-resolution saliency maps by modifying the standard backpropagation, thus emphasizing fine-grained input features that strongly activate the network, while not inherently focusing on any specific class. In contrast, Grad-CAM generates class-discriminative saliency maps by computing gradients of the target class logit with respect to the final convolutional layer feature maps; these gradients are then pooled to produce coarse spatial importance maps highlighting the regions most relevant to the class prediction (Selvaraju et al., 2020). Finally, Guided Grad-CAM combines the strengths of both methods, merging Grad-CAM class-discriminative localizations with Guided Backpropagation high-resolution details, thereby providing interpretable and precise visual explanations simultaneously (Selvaraju et al., 2020).

In the following sections, we describe the Guided Backpropagation, Grad-CAM, and Guided Grad-CAM computations, highlighting how the second poses a major limitation in the number of computed relevance scores depending on the structure of the ECGs as model input data, and thus the model interpretability. Furthermore, a new metric termed *lead importance* is introduced to assess which leads contain, more often than others, features that are ranked as important in the decision making process of the network.

#### 2.4.1 Guided backpropagation

Guided Backpropagation is a gradient-based approach to generate saliency maps that measure the relevance of each input feature in **S***_r_*in the computation of a target output score *z_c_*. Specifically, the saliency map to interpret the logit *z_c_*obtained for an input signal **S***_r_*is defined as

(2) $$B_{c}^{{\text{S}}_{r}}=\frac{\partial z_{c}}{\partial S_{r}}=\frac{\partial z_{c}}{\partial X_{0}},\;c=0,\ldots ,C-1,$$

where each gradient is computed by recursively applying the chain rule from the output to the input layer, modifying the standard backpropagation computations. Indeed, in standard backpropagation, the gradient with respect to the feature map **X***_i−_*_1_ – i.e., one factor in the chain rule – is given by


$$\frac{\partial z_{c}}{\partial X_{i-1}}=\frac{\partial X_{i}}{\partial X_{i-1}}\cdot \frac{\partial z_{c}}{\partial X_{i}},$$

which is modified in Guided Backpropagation into

(3) $$\frac{\partial z_{c}}{\partial X_{i-1}}=\{\begin{array}{ll} \frac{\partial X_{i}}{\partial X_{i-1}}\cdot \frac{\partial z_{c}}{\partial X_{i}}\cdot 1\left(\frac{\partial z_{c}}{\partial X_{i}}>0\right) & \text{if layer }i\text{ is a ReLU},\\ \frac{\partial X_{i}}{\partial X_{i-1}}\cdot \frac{\partial z_{c}}{\partial X_{i}} & \text{otherwise}, \end{array}$$

where *·* denotes the element-wise product. The gradient of the ReLU-activated output **X***_i_* with respect to the input **X***_i_*_−1_ is


$$\frac{\partial X_{i}}{\partial X_{i-1}}=\cdot 1\left(X_{i-1}>0\right),$$

which holds for both standard backpropagation and its modified version. In Guided Backpropagation, as a consequence of Equation 3, whenever a ReLU is encountered, the backward flow of negative gradients is suppressed, only allowing positive gradients to flow through, thereby focusing on features that positively influence the network activation, as originally proposed in (Springenberg et al., 2015).

Notice that, as a result of Equation 2, a relevance score is obtained for each input feature in the reshaped ECG signal, or equivalently, $S_{r},\;B_{c}^{S_{r}}\in {\mathbb{R}}^{D}$ always have the same dimensionality regardless of the ECG formatting.

#### 2.4.2 Grad-CAM

Grad-CAM is a gradient-based explainability method designed to identify the regions of the input that contribute most to a specific class prediction. It does so by establishing a connection between the class-specific logit *z_c_*from the final fully connected layer and the maps $x_{ℒ}^{j}$, $j=1,\ldots ,M_{ℒ}$, produced by the last convolutional layer, extracting saliency map $C_{c}^{S_{r}}$ as


$$C_{c}^{S_{r}}=ReLU\left(\sum_{j=1}^{M_{ℒ}}\alpha_{c}^{j}\cdot x_{ℒ}^{j}\right),\;c=0,\ldots ,C-1,$$

where $x_{ℒ}^{\text{j}}$ denotes the *j*-th feature map at the last convolutional block, and the weight $\alpha_{c}^{j}$ is defined as:


$$\alpha_{c}^{j}=\frac{1}{\left|\left\{f\in x_{ℒ}^{j}\right\}\right|}\sum_{f\in x_{ℒ}^{j}}\frac{\partial z_{c}}{\partial f}.$$

The weights $\alpha_{c}^{j}$ are obtained by applying GAP to the gradients of the target logit *z_c_*with respect to each activation *f* in the feature map $x_{ℒ}^{j}$ (Selvaraju et al., 2020). This weighting scheme captures the importance of each feature map in predicting class *c*, while the subsequent ReLU nonlinearity filters out negative influences, ensuring the final saliency map highlights only features that positively contribute to the class decision.

It is important to highlight that the number of features in the feature maps, and therefore the number of scores in the saliency maps, can differ from the total number of input features *L · T*, that is, the number of time step per number of leads. Indeed, as discussed for every FCN, the dimensionality of feature maps varied with the ECG shaping and with the stride hyperparameter. For this reason, not all of the feature maps were suitable for explaining all the values in the ECGs.

Specifically, when using multi-channel FCNs with one-dimensional convolutional kernels of Section 2.2.2, the network extracted one-dimensional feature maps with one feature per time step, i.e. $x_{ℒ}^{j}\in {\mathbb{R}}^{T},\;\forall \;j=1,\ldots ,M_{ℒ}$. Their weighted sum resulted in saliency maps that assigned one relevance score per time step, namely $C_{c}^{S_{r}}\in {\mathbb{R}}^{T}$, collapsing the lead dimension and thus preventing separate assessment of each of the 12 leads’ contributions to the classification.

Conversely, in the single-channel FCN configuration (Section 2.2.1), where all leads were stacked sequentially to form a univariate time series, the feature map dimensionality matched the input dimensionality. However, the use of a stride greater than 1 lead to extracting feature maps shorter than the full length of the stacked input. This discrepancy reduced the number of relevance scores compared to the original input length, necessitating interpolation to restore matching dimensions. Such interpolation risks losing important fine-grained information about the model’s decision function.

Therefore, to obtain saliency maps that are both high-resolution and able to discriminate contributions from each lead at every time step, only the single-channel FCN with 2D filters with a stride of 1 provided a suitable framework where $C_{c}^{S_{r}}\in {\mathbb{R}}^{T\times L\times 1}$ matched the dimension of the ECG signal. This approach preserved both temporal and lead-specific resolution, enabling more interpretable and granular explanations of the model predictions.

#### 2.4.3 Guided Grad-CAM

Guided Grad-CAM is a XAI method that combines the localization capabilities of Grad-CAM with the fine-grained sensitivity of Guided Backpropagation to generate high-resolution saliency maps (Selvaraju et al., 2020), inheriting the spatial precision of Guided Backpropagation and the class-discriminative properties of Grad-CAM. Guided Grad-CAM integrates these two methods by performing an element-wise product between the Guided Backpropagation gradients and the Grad-CAM saliency map, meaning that the final saliency map $M_{c}^{S_{r}}$ computed for instance **S***_r_*and logit *z_c_*is


$$M_{c}^{S_{r}}=B_{c}^{S_{r}}\cdot C_{c}^{S_{r}},\;c=0,\ldots ,C-1,$$

which can be computed when $B_{c}^{S_{r}}$ and $C_{c}^{S_{r}}$ have the same dimensionality, a condition that was met only when the inputs were fed as images.

In this work, we focused on Guided Grad-CAM saliency maps of correctly classified test samples, using the absolute values of the scores. This approach was motivated by the findings of Suh et al. (2024) in the context of pathological ECG classification, where the regions highlighted by the absolute Guided Grad-CAM scores were shown to well correspond with features identified by medical experts as clinically relevant. As a preliminary step to interpreting the network’s decisions, the ECGs within a given region were partitioned into clusters as described in Section 2.4.4, in order to refine the analysis to account for morphological differences in same-class data, possibly affecting highlighted ECG segments in the saliency maps. Subsequently to clustering, the saliency maps were visually inspected by the expert in ECG generation through computational methods responsible of the data generation, to understand whether the features driving the network’s choices could be linked to a physiological interpretation. Further considerations were made to assess the effectiveness of the Guided Backpropagation and Grad-CAM methods in highlighting pathology-related patterns.

#### 2.4.4 Physiological interpretation of XAI results

To assess the relationship between ECG morphological features highlighted by the XAI methods and the underlying EP activity, a qualitative visual analysis was conducted. This analysis aimed to correlate the evolution of the transmembrane voltage signal in the virtual heart model with the corresponding ECG segments identified by the XAI techniques. Since the 24 anatomical regions were defined *a priori*, substantial variability in ECG morphology was observed among signals associated with the same region, making it challenging to establish a generalized correlation between highlighted ECG features and specific anatomical locations. These variations were also reflected in the XAI outputs. To address this limitation, we applied clustering within each region to group ECGs with similar morphological patterns, thereby enabling a more consistent and interpretable mapping between ECG features and AP locations.

We employed the k-medoids clustering algorithm in combination with the Dynamic Time Warping (DTW) distance (Müller, 2007) to group signals with similar shapes, regardless of temporal shifts. This approach allowed us to cluster ECGs based exclusively on their morphological features, consistently with the focus of the XAI techniques, which highlight relevant regions of the waveform without considering absolute timing. K-medoids selects a real ECG signal as the cluster representative, preserving physiological interpretability while also reducing the computational cost associated with centroid estimation (Izakian et al., 2015). For each anatomical region, we explored a range of cluster numbers *k ∈ {*2,3,4*}*, and selected the optimal *k* by maximizing the average silhouette score (Belyadi and Haghighat, 2021), which quantifies both intra-cluster similarity and inter-cluster separation.

#### 2.4.5 Lead importance

The XAI method used in this work produces a relevance score for each time step and each lead of the input ECG, resulting in a normalized importance map across all leads. While originally intended to explain individual classifications, this map can also be exploited to uncover global morphological patterns and guide the development of future diagnostic strategies. Specifically, it enables the identification of the most influential leads, those whose variations most strongly contribute to the model predictions. To quantify this, we define an *index of lead importance* that summarizes the information extracted from the XAI maps across the test set.

Intuitively, for a sample correctly classified as belonging to class *c*, the higher the relevance scores associated with a given lead, the greater its contribution to the classification decision. To evaluate this systematically across all correctly classified samples, we define the lead importance for each lead *l* = 1*,…,L* and class *c* = 0*,…,C −* 1 as:


$$LI_{l}^{c}=\frac{\sum_{S_{r}\in C_{c}}\sum_{t=1}^{T}{\left(M_{c}^{S_{r}}\right)}_{tl}}{\sum_{l=1}^{L}\sum_{S_{r}\in C_{c}}\sum_{t=1}^{T}{\left(M_{c}^{S_{r}}\right)}_{tl}},$$

where ${\left(M_{c}^{S_{r}}\right)}_{tl}$ denotes the absolute value of the Guided Grad-CAM relevance score at time step *t* in lead *l*, for the sample **S***_r_*belonging to the set *C_c_*of test samples correctly assigned to class *c*. The results of this analysis are presented in Section 3.2.

## 3 Results

All of the DL models described in Sections 2.2.1-2.2.3 were trained and then fine-tuned at the last layers to further refine the higher-level extracted representations. The ECG dataset was split into training, validation, and test sets with a proportion of (75, 15, 10)%. Additionally, two ablation studies were performed to assess the degradation in model performance on more challenging datasets derived from the original pool of 8842 APs. Related findings are discussed in Section 3.1.1. In all instances, the validation loss was used to select the best model and trigger early stopping, both during training and fine-tuning. The latter consisted of adopting a lower learning rate and freezing all weights except those of the last convolutional block and dense layer. Details on the hyperparameters of every FCN are reported in the **Supplementary Materials**.

### 3.1 Classification performance

Following training and fine-tuning on the (75, 15, 10) split, both the multi-channel FCN and the single-channel FCN with 2D kernels achieved comparable accuracies on the test set – 95.48% and 94.12%, respectively. In contrast, treating ECGs as a stack of leads resulted in a lower performance of 91.98%.

Given its suitability for XAI analysis and the high accuracy results, the 2D-FCN was selected as the reference network for all subsequent analyses. For the 2D-FCN, the macro-F1 score and the mean class-wise recall (balanced accuracy) amounted to 93.67% and 93.05%, respectively. Sensitivity and specificity were computed for this model and are reported in **Table 1**. Optimal classification is achieved for most regions, including most of the septal ones.

**Table 1:** Class-wise specificity, sensitivity, and cardinality.

Class	Specificity (%)	Sensitivity (%)	Train cardinality	Test cardinality
0	99.64	88.52	457	61
1	99.27	96.72	457	61
2	99.76	88.37	316	43
3	99.39	93.94	490	66
4	99.52	96.36	415	55
5	99.28	98.25	424	57
6	99.77	98.25	213	28
7	99.88	95.00	146	20
8	99.89	92.86	110	14
9	99.77	83.33	140	18
10	100.00	81.81	84	11
11	99.88	88.24	126	17
12	100.00	95.92	367	49
13	99.76	98.15	402	54
14	99.76	100.00	322	43
15	99.88	100.00	327	44
16	99.03	98.28	434	58
17	99.76	91.30	344	46
18	99.89	100.00	113	15
19	100.00	93.94	251	33
20	100.00	95.45	164	22
21	99.88	100.00	149	20
22	99.88	79.17	184	24
23	99.77	84.62	196	26

Reduced sensitivity was displayed in regions 0, 2, 9, 10, 22 and 23, with the latter four corresponding to the mid and mid-ventricular segments in the bi-ventricular mesh, as displayed in **Figures 2**, **3**. In these cases, false negatives were frequently classified into the anatomically corresponding basal region within the same rotational coordinate interval. This misclassification pattern is attributable to the lower density of sampled points in the mid-ventricular region, which resulted from physiological constraints imposed by the computational model used for data generation, in conjunction with class stratification being preserved during the data split.

Visual inspection of the data misclassified by the single-channel FCN with 2D kernels revealed that most errors occurred, understandably, for ECGs corresponding to APs located near the boundary between two or more adjacent regions, as illustrated in **Figure 4A**. Notably, out of 52 misclassified test samples, 47 had the second largest predicted probability corresponding to the true class, yielding a top-2 accuracy of 99.44%.

**Figure 4:**
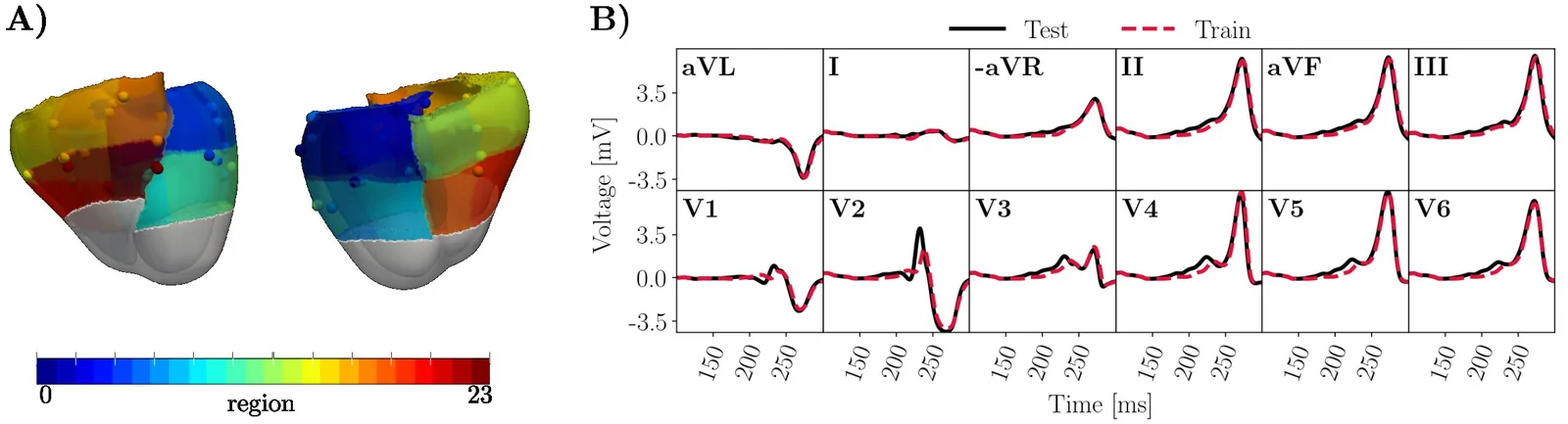
Data misclassified by FCN in Section 2.2.3. **(A)** APs of misclassified data are plotted with the color of the region they have been assigned to. **(B)** Comparison of a misclassified test sample (continuous) with a correctly classified training sample (dashed) assigned to the same class.

A specific analysis was performed to evaluate the ability of the 2D FCN to identify the septal antegrade region, located near the His bundle and the atrioventricular (AV) node, which are traditionally difficult to localize (Arruda et al., 1998; El Hamriti et al., 2023). To this end, regions 3, 5, 9, and 11 were grouped together due to their proximity to the His bundle, while regions 1, 2, 7, and 8 were grouped to encompass the medial-posterior septum. The first set of regions achieved a sensitivity of 94.94% and a specificity of

98.35%, whereas the latter set achieved 95.65% sensitivity and 99.20% specificity.

#### 3.1.1 Dataset ablation

Considering the dense sampling of APs in our dataset, and the possible similarity between ECGs generating by neighboring APs, we additionally investigated the robustness of the proposed classifier under more challenging datasets designed to reduce the spatial proximity between training and test samples. Two alternative dataset splits were considered.

In the first, referred to as the *buffered* split, 500 APs were randomly selected to constitute the test set. Subsequently, all APs lying within three mesh edges of any test AP were excluded from the training and validation sets, yielding a buffer of three edges around any test AP. Final training and validation sets amounted to 3309 and 828 samples, respectively.

In the second split, denoted as *kmeans*, APs were first clustered according to their Cartesian coordinates using *k*-means clustering algorithm. The number of clusters was chosen to be substantially larger than the number of anatomical regions considered for classification, so that each cluster remained spatially localized and covered a small portion of a region. Namely, 240 clusters were obtained, out of which 24, one for each classification region, were assigned to the test set, finally comprising 999 samples. After removing further APs, corresponding to those within three mesh edges of the test set, as per the *buffered* split, the remaining samples were divided into training (5108 samples) and validation (1277 samples) sets. This test effectively aims at blinding the network from entire sub-regions.

The proposed 2D-FCN achieved test accuracies of 89.20% and 87.09% on the *buffered* and *kmeans* splits, respectively. The corresponding macro-F1 scores were 87.57% and 84.28%, while the mean class-wise recalls amounted to 86.69% and 84.02%. Notably, the top-2 accuracy of both spatially blocked splits is comparable to that of the (75, 15, 10) split, amounting to 98.00% in the first case and 99.30% in the second.

Interestingly, in most cases, the AP closest to a test sample in mesh space did not correspond to its nearest ECG neighbor under the DTW distance. Consequently, increasing the spatial separation between APs does not eliminate highly similar ECGs across the dataset.

#### 3.1.2 Comparison with the EASY-WPW and Arruda algorithms

We compare the performance of the EASY-WPW and Arruda DT algorithms with our 2D-FCN trained over the entire dasates, by regrouping the ECGs according to the anatomical regions defined by each DT method, and we manually classify the ECGs following the corresponding DT rules. A McNemar’s exact test was finally performed. On the EASY-WPW regions, the FCN correctly classified 100% of the subset samples, whereas the DT achieved only 64% accuracy (p-value = 7.45 *·* 10*^−^*^9^). Similarly, the network demonstrated superior performance on the Arruda regions, attaining 97.33% accuracy compared to 37.33% for the Arruda algorithm (p-value = 3.41 *·* 10*^−^*^12^).

### 3.2 Physiological interpretation of XAI results

Saliency maps derived from all three evaluated XAI techniques revealed consistent identification of morphologically relevant features across the 12-lead ECG that serve as biomarkers in WPW syndrome, corresponding to the anatomical location of the AP across various cardiac regions, though the extent and localization of activations varied across clusters (**Figure 5**). Within the saliency representations, ECGs associated with right ventricular APs formed distinct clusters characterized by stable morphological patterns, whereas left ventricular APs exhibited greater heterogeneity (we referred to examples of saliency maps with Guided Grad-CAM for the right and left ventricles in **Figures 6**, **7**). Despite inter-cluster variability, all XAI techniques consistently highlighted key morphological features - most notably the QRS complex and delta wave - across all cases, underscoring their diagnostic relevance in identifying AP-mediated conduction abnormalities.

**Figure 5:**
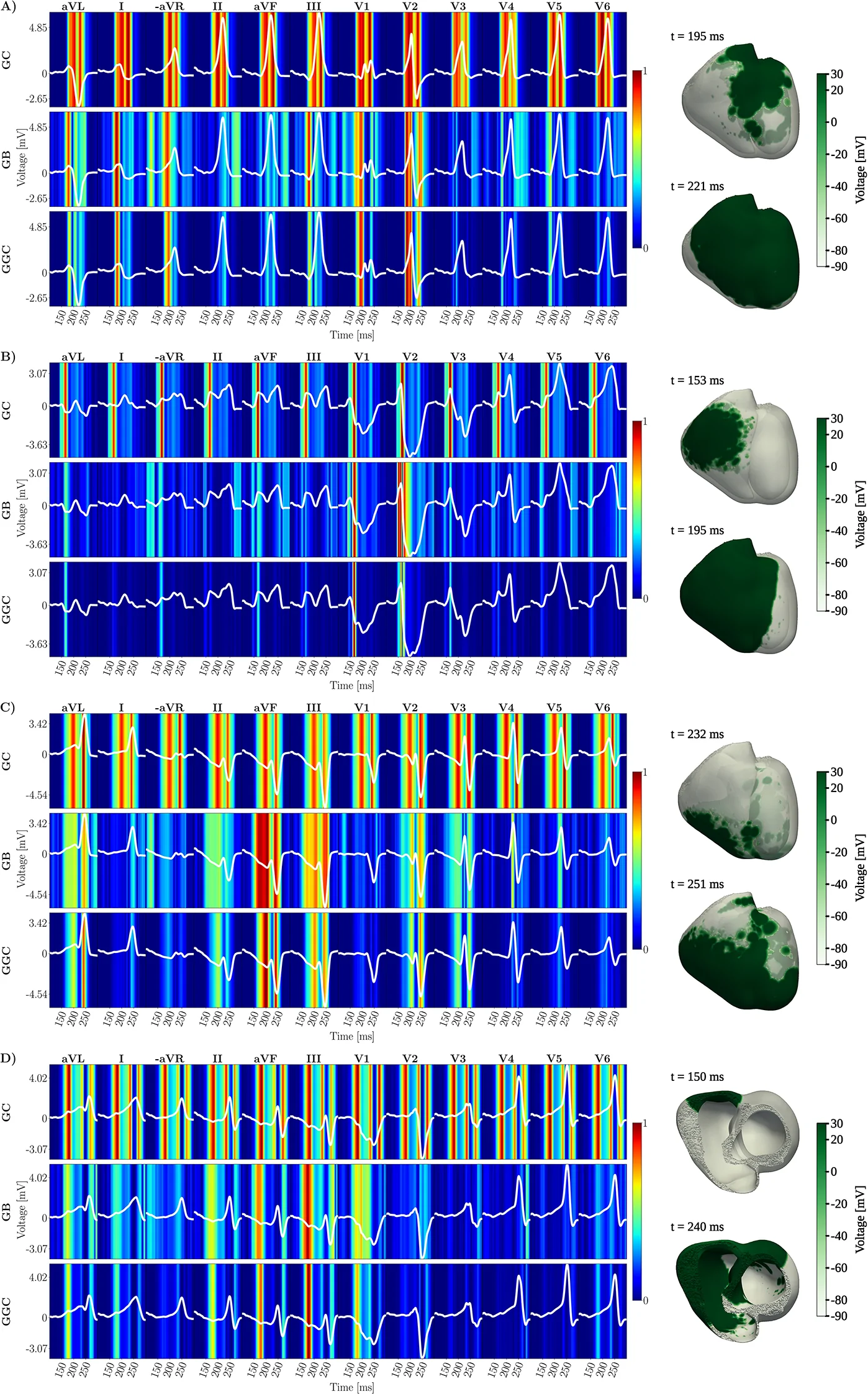
Grad-CAM (GC), Guided Backpropagation (GB), and Guided Grad-CAM (Guided GB) saliency maps, and activation patterns across ECG leads vary with the anatomical location and conduction behavior of APs. **(A)** AP contributing to the ongoing wavefront highlights features in the QRS complex in multiple leads, especially in aVL, V1, and V2. **(B)** Right ventricular AP demonstrates localized activation in leads V1 and V2, consistent with activation disruptions from the RV free wall. **(C)** AP initiating early ventricular activation produces diffuse explainability across several leads. **(D)** AP in the RV free wall outside the His-Purkinje system reveals two distinct ECG regions: the delta wave and His-Purkinje capture.

**Figure 6:**
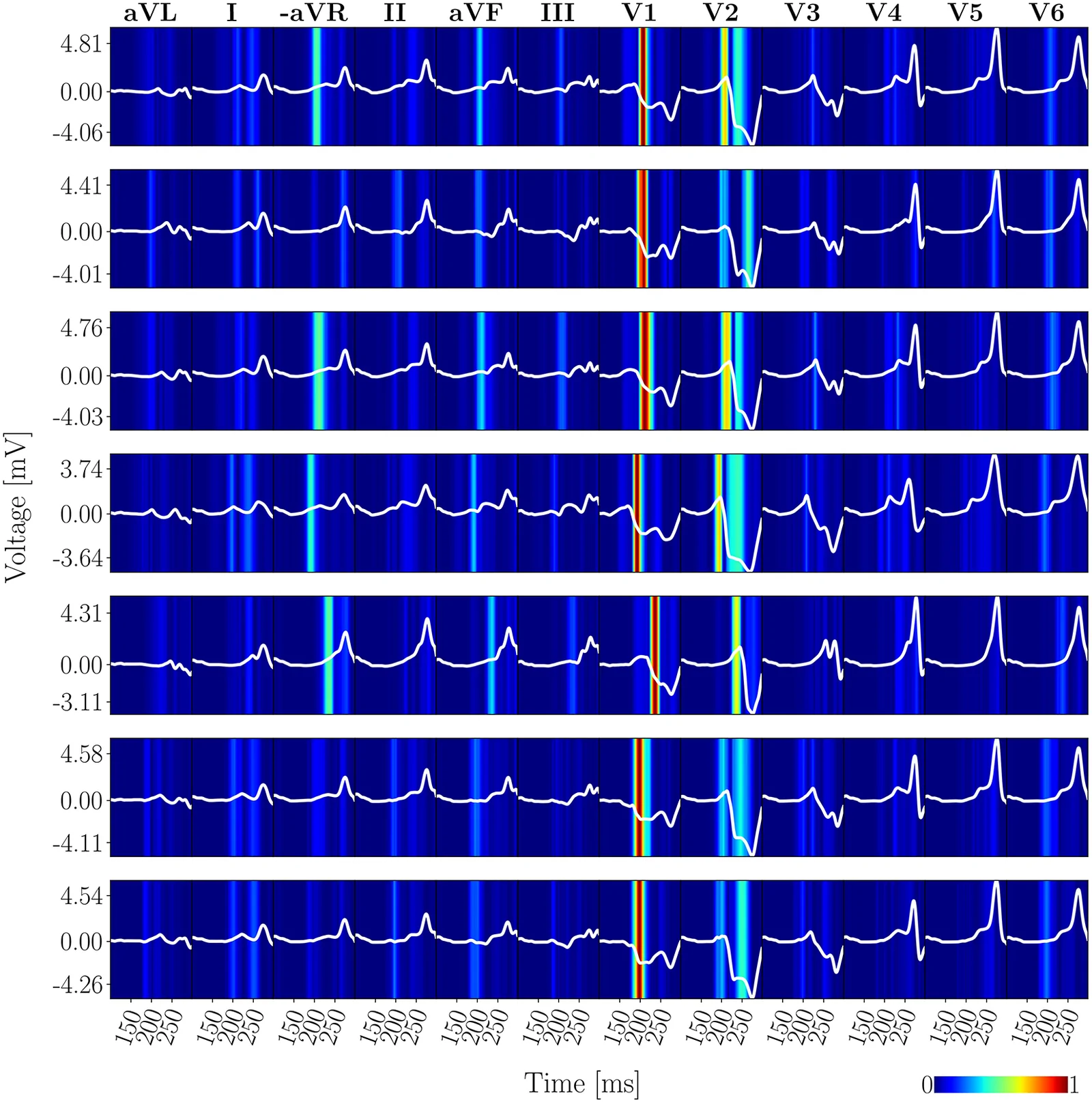
Example of ECG variation for a cluster in the right ventricle (region 14), and corresponding saliency maps computed with the Guided Grad-CAM.

**Figure 7:**
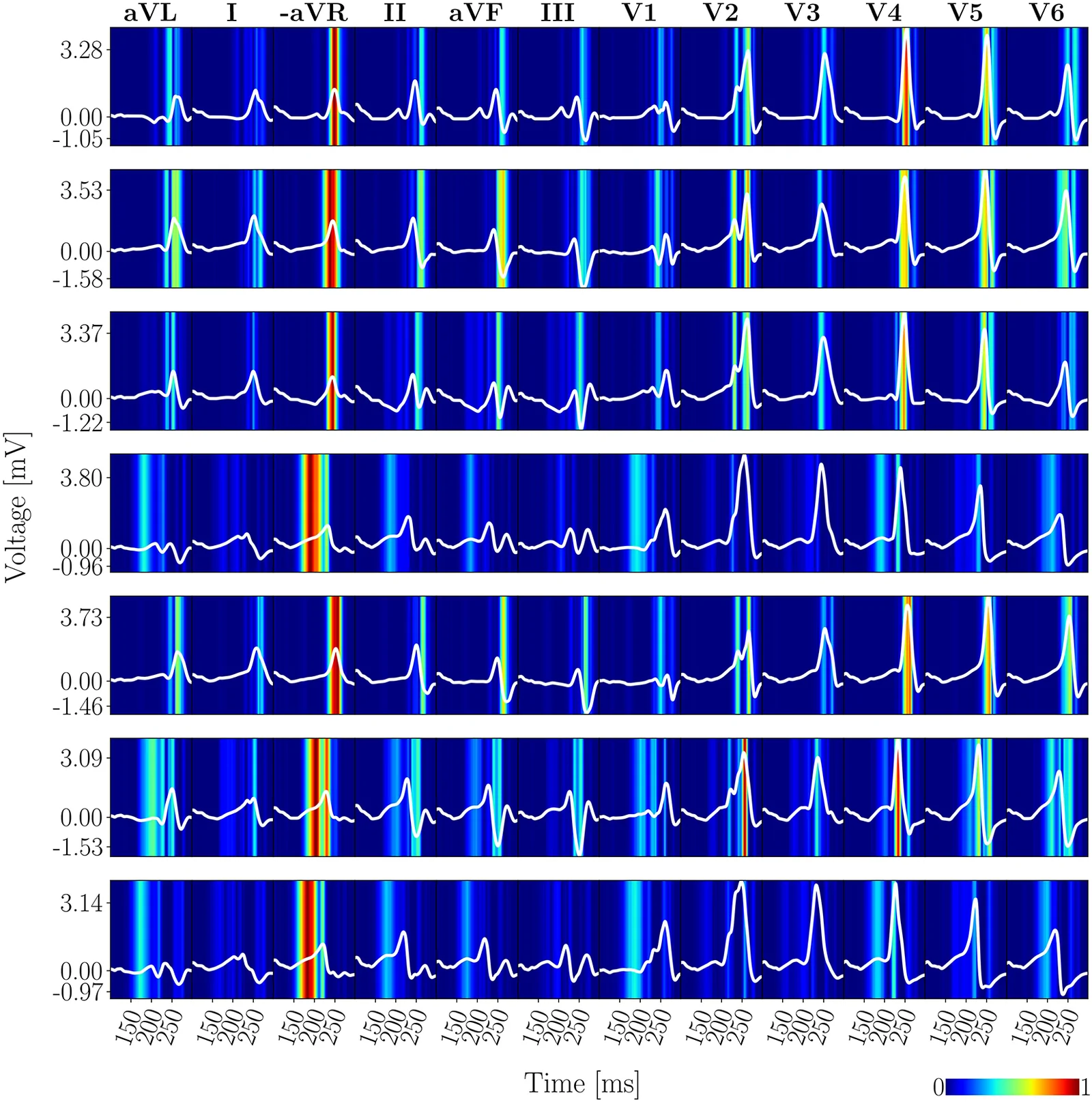
Example of ECG variation for a cluster in the left ventricle (region 1), and corresponding saliency maps computed with the Guided Grad-CAM.

More specifically, across all clusters, Grad-CAM produced broad activations spanning multiple leads – particularly within the QRS complex – reflecting the generally altered ventricular activation patterns induced by the AP (**Figure 5**). In contrast, Guided Backpropagation demonstrated greater spatial selectivity, emphasizing specific leads while still targeting similar temporal regions of the signal. The Guided Grad-CAM approach, which combines the coarse localization of Grad-CAM with the fine-grained sensitivity of guided backpropagation, yielded the most precise identification of diagnostically relevant features. This method produced highly localized activations that aligned closely with the underlying AP morphology and its electrophysiological impact on the 12-lead ECG.

Further physiological analysis and interpretation revealed distinct patterns of XAI activation not always aligned with standard diagnostic heuristics: clinically prioritized features (e.g., the inferior delta wave) were occasionally underweighted despite correct classification, while the networks also emphasized non-canonical features (e.g., inferior QRS fragmentation) not typically considered diagnostic. However, a relation between XAI highlighted areas and transmembrane voltage propagation was identify, depending on the anatomical location, timing, and the conduction behavior of the AP. When the AP was positioned and timed such that it contributed to the ongoing wavefront of cardiac propagation (**Figure 5A**), XAI methods reliably highlighted corresponding features in the onset of the QRS complex across multiple leads, most notably in aVL, V1 and V2. In right ventricular APs, localized activations appeared predominantly in leads V1 and V2 (**Figure 5B**), consistent with expected disruptions originating from the right ventricular free wall. Conversely, when APs initiated premature ventricular contraction from a site not integrated into the normal conduction pathway, the XAI techniques highlighted extended segments across several leads (**Figure 5C**), indicating a more diffuse impact on cardiac morphology. This is further shown with an AP located in the RV free wall outside of the His-Purkinje system (**Figure 5D**). The XAI techniques detect two primary portions at different times of the ECG, corresponding to the delta wave and directly during His-Purkinje capture. Collectively, these patterns highlight how XAI-derived saliency maps capture both spatial and temporal characteristics of AP-mediated conduction, reflecting underlying electrophysiological mechanisms observed in WPW syndrome.

### 3.3 Lead importance

Following the XAI results and interpretation, we focused on investigating the Guided Grad-CAM method to further quantify which ECG leads most frequently contained features deemed influential by the XAI method in the model’s class predictions. The lead importance *LI_l_^c^* in (Schiavone et al., 2024) is reported for each lead-class pair as a heatmap in **Figure 8A**. For completeness, a study on the robustness of lead importance with respect to lead permutation in the construction of the ECG image is reported in the **Supplementary Materials**. The distribution of lead contributions was non-uniform across regions, indicating that specific leads play a more dominant role in guiding classification. Among these, leads V2, aVR, aVL, and V1 consistently exhibited the highest importance across multiple anatomical classes. The frequency with which these leads emerged as important after training on ECG images built from lead permutations is reported in the **Supplementary Materials**.

**Figure 8:**
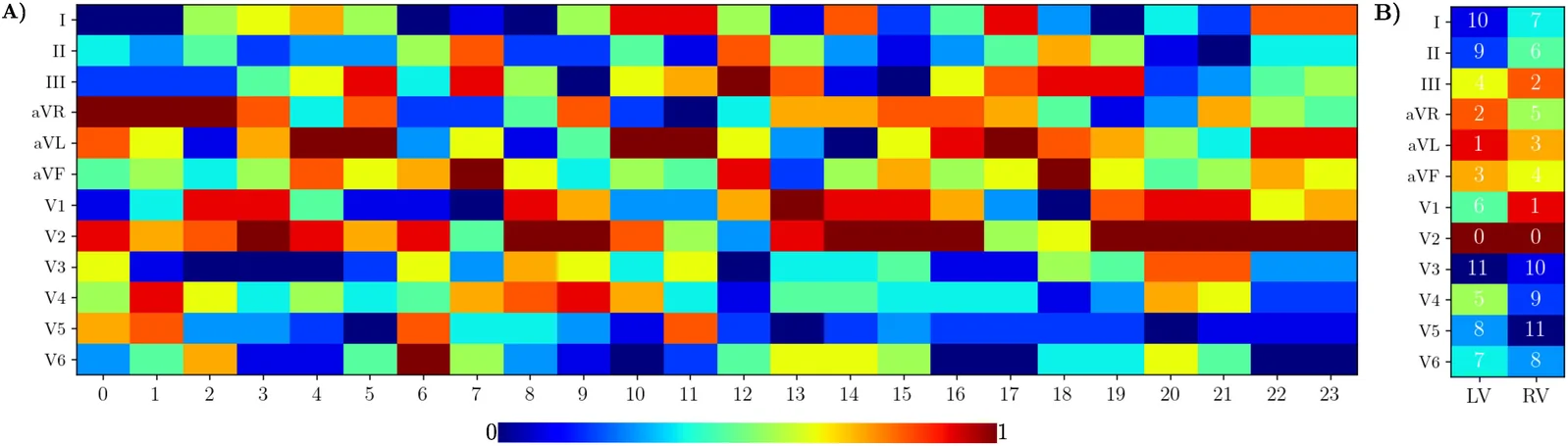
Lead importances according to (Equation 5) under the model trained with leads ordered as I, II, III, aVR, aVL, aVF, V1-V6. **(A)** Lead importance for each of the 24 regions. **(B)** Lead importance grouping LV and RV signals, ranked from most (0) to least (11) important.

To enable regional comparison, signals were further grouped by ventricular origin of the AP, and aggregated lead importance indices were computed for left and right ventricular APs and displayed in **Figure 8B**. These ventricular-level summaries highlight consistent intraventricular patterns of lead relevance, with V2 remaining the most important lead for both right and left ventricular AP, immediately followed by lead V1 in the RV, and aVR and aVL in the LV.

## 4 Discussion

### 4.1 Classification performance

In this work, we investigate a promising deep learning–based model for the localization of APs. Among the three evaluated architectures, two achieved accuracies exceeding 94%, an improvement compared to most previously reported AP localization algorithms, including both DT- and ML-based approaches (Arruda et al., 1998; Pambrun et al., 2018; Nishimori et al., 2021; Senoner et al., 2021; El Hamriti et al., 2023; Yahyazadeh et al., 2024). Even under spatially-blocked splits the model proved able to extrapolate and maintain good performance over the 24 anatomical regions. The performance improvements over existing DT-based algorithms were further analyzed by comparing the FCN model with manual classifications of our ECGs using two representative DTs. The FCN resulted in 100% accuracy on the EASY-WPW regions and 97.33% on the Arruda regions, scoring higher accuracy than the two DT-based methods, which achieved 64.00% and 37.33% overall accuracy, respectively.

Although the DT-based algorithm in the work of Khalaph et al. (2025) achieved a marginally higher accuracy of 97%, it was restricted to 12 manually defined regions describing only the rotational position of the AP. In contrast, our approach considers 24 classes, introducing a longitudinal subdivision that enhances the spatial resolution of AP localization. Furthermore, the classification regions were defined using standardized UVC coordinates, ensuring reproducibility and anatomical consistency across subjects.

However, these results should be interpreted with caution. The analysis is based on a limited, synthetic dataset derived from a single subject. As such, the observed differences may not generalize to broader or real-world settings. Overall, these findings provide preliminary evidence that the proposed approach may yield improved performance, but further validation on larger, more diverse datasets is required.

Three CNN architectures sharing the same core structure but differing in input dimensionality were implemented and systematically evaluated to optimize localization performance. To mitigate data scarcity and enhance training variability, a virtual dataset was used, previously generated by Gillette et al. (2025) using a cardiac digital twin, simulating ECGs corresponding to APs at multiple physiological cardiac locations. The ECGs are intended as pre-ablation, where no further ablation lesions can influence the cardiac EP. This strategy effectively overcomes a key limitation of previous studies (Senoner et al., 2021; Middlehurst et al., 2024), where the small number of available ECGs constrained both the training set size and the number of anatomical regions that could be defined as classes. By generating synthetic ECGs through the digital twin, our dataset is rich in diversity, which may support improved generalization of the FCN to unseen data.

To the best of our knowledge, this study provides the first rigorous analysis of how different input data structures affect ECG-based classification, explicitly considering lead differentiation and the identification of lead-specific importance during model inference. Our results demonstrate that optimal performance with the FCN is achieved when both single-lead information and temporal dependencies are explicitly modeled. This configuration is realized in the multi-channel FCN, where the feature maps capture lead-wise and temporal relationships for each lead, and in the image-like 2D FCN, where square kernels jointly encode lead (width) and temporal (height) dependencies. In contrast, the classifier based on a single stacked input of leads exhibited the lowest accuracy, reflecting the absence of explicit spatial and temporal characterization. Future improvements could involve the use of variable-length or dilated kernels across the lead stack, as proposed by Ayano et al. (2024).

### 4.2 Explainability

To ensure model transparency and physiological interpretability, three XAI techniques were implemented and rigorously evaluated. While not always corresponding to standard diagnostic heuristis, the resulting saliency maps demonstrated a strong correspondence between the model’s highlighted features and established electrophysiological patterns, confirming that the network localized APs based on physiologically meaningful signal propagation across the 12-lead ECG (**Figures 5**–**7**). These findings validate the internal consistency of the FCN and its ability to leverage morphological information in a manner coherent with known cardiac conduction mechanisms.

Model interpretability can be assessed at two complementary levels: individual predictions and aggregate population trends. Most existing explainable ECG classification studies (Jones et al., 2020; van de Leur et al., 2021; Le et al., 2022; Xie et al., 2024) have focused primarily on the former, visually inspecting the salient regions for single cases or diagnostic classes, often without quantifying broader lead-level trends across datasets. In contrast, the present study integrates both perspectives. Following methodologies similar to Ayano et al. (2024) and Agrawal et al. (2022), saliency maps were first analyzed qualitatively, then summarized quantitatively through the computation of a lead-importance index for each lead–class pair. This metric provides a reproducible, dataset-level measure of the relative influence of each lead in model decision-making, a quantity not formalized in previous works, but only qualitatively observed.

To ensure accurate and interpretable saliency mapping, the 2D-kernel FCN was used, as it guarantees that saliency maps match the input signal dimensions. Previous methods (Le et al., 2022; Ayano et al., 2024) processed each lead independently using 1D CNN backbones, combining the resulting feature maps through attention mechanisms or concatenation. While effective, this joint optimization introduces dependencies across leads, meaning that the relevance scores for a given lead cannot be considered fully independent. By contrast, processing the ECG as an image with a 2D FCN provides a straightforward and physiologically consistent framework for saliency analysis across all 12 leads, avoiding the added complexity of multi-backbone architectures.

Visual inspection of the saliency maps revealed that activation patterns varied systematically with AP location and conduction characteristics. For right ventricular pathways, saliency concentrated around precordial leads V1 and V2, while left-sided pathways were characterized by stronger activity in aVR and aVL. These trends are consistent with the spatial projections of ventricular depolarization on the standard 12-lead ECG. Quantitative analysis of lead importance further confirmed this pattern: leads V2, aVR, aVL, and V1 consistently emerged as the most influential across multiple anatomical classes. At the ventricular level, V2 remained the most important lead for both right and left APs, followed by V1 in the RV and aVR and aVL in the LV, albeit in varying order of relevance depending on the ordering of leads as columns of the ECG image.

The pronounced importance of the augmented Goldberger leads (aVL, aVF, aVR) compared with the standard limb leads (I, II, III) likely stems from their unipolar configuration. Unlike bipolar limb leads, which measure the potential difference between two electrodes, Goldberger leads reference the Wilson central terminal, a composite average of the other limb electrodes. This effectively amplifies signal magnitude and enhances sensitivity to subtle morphological variations, which deep learning models can exploit more efficiently. Consequently, the network may capture electrophysiological nuances in these leads that are less discernible in standard limb leads.

Importantly, the ranking of influential leads derived from the XAI analysis aligns closely with independent sensitivity studies. Gillette et al. (2025) similarly identified V2 as the most sensitive lead for accessory pathway localization in the same dataset. This convergence supports the physiological validity of the explainability framework and suggests that the FCN internal representations reflect true cardiac conduction dynamics rather than spurious correlations. Nonetheless, this observation should be interpreted with caution: the prominence of V2 may be partially influenced by the specific heart orientation and electrode positioning in the subject used to generate the data. Expanding the dataset to include a broader range of anatomical and geometric configurations could provide further insight into the generalizability of these findings.

Finally, by identifying a subset of leads with high diagnostic relevance, the lead-importance framework provides practical insights for future work in model design. Training lighter DL architectures on low dimensional input centered on these key leads presents the potential to preserve diagnostic accuracy while improving computational efficiency. More broadly, the proposed approach illustrates how explainability metrics can bridge data-driven inference and physiological understanding, ensuring that model predictions remain interpretable and clinically grounded.

## 5 Limitations

The main limitation of this work lies in the dataset employed to train the FCN. While rich in terms of the number of ECG traces and possible variability depending on the AP position, it was restricted to a single subject. The high number of ECGs might improve the network’s ability to learn ECG variations in the single cardiac model, acting as a sort of interpolation method, thus strongly limiting possible natural inter-patient variability. This hinders the generalization ability of the network to unseen data coming from cardiac digital twins of other patients and clinical data. The scope of future work will be to augment this dataset, including more subjects, and to test the obtained network on real clinical data.

The dataset used for training and evaluation was generated from a single subject’s anatomy, which constrains the anatomical variability captured in the study and may influence lead-specific importance patterns, such as the prominent role of V2. Although the virtual dataset created via a cardiac digital twin increases the diversity of training examples, it cannot fully reproduce the anatomical and electrophysiological heterogeneity present in a broader population. Expanding the model to real patient data will be essential to validate generalizability and confirm the physiological relevance of observed lead-importance trends. In addition, the present model was developed for single manifest antegrade accessory pathways with clear pre-excitation on the surface ECG. Clinically challenging scenarios such as concealed accessory pathways, and intermittent pre-excitation were outside the scope of this study and will require dedicated modeling and validation in future work.

Further limitations arise from the limited theoretical foundation of *post-hoc* explainability methods in the context of time series data. While interpreting saliency maps for image data is relatively straightforward due to the explicit semantics (e.g., edges, colors), physiological time series such as ECGs are not yet fully understood, and the relationship between the ECG and the underlying electrophysiology remains unclear. Moreover, even with 2D-kernel FCNs, the relevance scores for individual leads may be influenced by interactions with other leads, limiting the independence of lead-specific interpretations. No formal mathematical framework currently exists to validate the physiological significance of saliency map features, making expert clinical evaluation necessary. These limitations will be addressed in future work to strengthen both the robustness and interpretability of the model.

## 6 Conclusions

In this work, we present the first DL-based method, implemented as a FCN, for the localization of antegrade APs within the ventricles. Our approach shows improved performance over existing DTand ML-based techniques in both accuracy and spatial precision, enabled by a higher number of anatomical classes. XAI techniques were applied to enhance model transparency, revealing a clear correspondence between predictions and underlying electrophysiology. A new index was introduced to identify the most influential lead in AP classification, exposing potential limitations in current localization strategies.

However, these findings are based on a synthetic dataset derived from a single subject and should therefore be interpreted as preliminary. Further validation on larger, more diverse cohorts and real-world data is required to assess the robustness and clinical applicability of the proposed approach. Overall, the results suggest that explainable deep learning could provide a useful framework for physiologically informed AP localization, with potential to support clinical decision-making and improve diagnostic workflows.

## Data Availability

The original contributions presented in the study are included in the article/**Supplementary Material**. Further inquiries can be directed to the corresponding author.
