# Supplementary material for: Explainable deep learning-based classification of Wolff-Parkinson-White electrocardiographic signals
Source: Front Physiol. 2026 Sep 23;17:1855555. doi: 10.3389/fphys.2026.1855555 (PMC13642006; doi:10.3389/fphys.2026.1855555)
Supplement: Supplementary file 1 [file DataSheet1.pdf]

Supplementary Material

1 SELECTED VALUES FOR HYPERPARAMETERS

The three Fully Convolutional Network (FCN) architectures were trained with the hyperparameter settings summarized in Table S1, resulting in 1161016, 531000, 3996120 trainable parameters, respectively. The best performance on the test set was yielded by the FCN with the lowest complexity and, therefore, the least expensive to train.

Layer	Hyperparameter	FCN 2.2.1	FCN 2.2.2	FCN 2.2.3
Layer 1	Num. filters	96	96	128
	Kernel size	100	9	9
	Stride	10	1	1
Layer 2	Num. filters	256	256	192
	Kernel size	20	9	9
	Stride	1	1	1
Layer 3	Num. filters	128	128	128
	Kernel size	20	9	9
	Stride	2	1	1

Table S1. Hyperparameters of the three FCN variants for each layer. Entries in green refer to values obtained from tuning, while yellow highlights the prescribed stride hyperparameter.

2 RELATIONSHIP BETWEEN DATASET REGIONS AND DIAGNOSTIC TREE REGIONS

Given the differing definitions of anatomical regions within the ventricles used in electrocardiogram (ECG) classification, we compared our Deep Learning (DL)-based model with classical Diagnostic Tree (DT) approaches by regrouping our regions to align with those defined by the DT algorithms. Specifically, our 24 reference regions were mapped to the EASY-WPW algorithm in El Hamriti et al. (2023), as shown in Table S2, and to the DT algorithm proposed by Arruda et al. (1998), as shown in Table S3. The mapping was manually constructed based on the descriptions provided in the reference works for EASY-WPW and Arruda – positioning the ventricles in a 60 degrees left anterior oblique view and following the authors reported instructions. Since our 24 regions include a subdivision along the longitudinal ventricular axis (apico-basal), rather than only a rotational subdivision as in the DT algorithms, the mapping was defined based on the superior regions, i.e., those closest to the ventricular base. The inferior regions were then grouped with their corresponding superior regions according to rotational position. The regrouping of the regions were revised and approved by a cardiologist expert in Wolff-Parkinson-White (WPW) diagnostic.

A random subset of 75 ECGs from the test set was selected for classification using the EASY-WPW and Arruda DT algorithms. The corresponding ground-truth ECG labels, based on the DT region subdivisions, were automatically derived using the Universal Ventricular Coordinate (UVC) framework. For each DT region, a corresponding UVC boundary interval was defined, and any ECG generated by an accessory pathway (AP) located within that interval was assigned to the corresponding DT region. A graphical representation of this UVC-based mapping is provided in Figure S1.

DT region	Dataset regions
MV-AL	$\{3, 9, 4, 10, 5, 11\}$
MV-PL	$\{0, 6, 1, 7, 5, 11\}$
MV-PS	$\{1, 7, 2, 8\}$
TV-AL	$\{13, 19, 14, 20, 15, 21, 16, 22, 17, 23\}$
TV-PL	$\{12, 18, 13, 19\}$
TV-PS	$\{12, 18, 1, 7, 2, 8\}$
TV-AS	$\{17, 23, 2, 8, 3, 9, 4, 10\}$

Table S2. Map between our 24 anatomical regions and the 7 regions used by the EASY-WPW algorithm presented in El Hamriti et al. (2023).

DT region	Dataset regions
LAL	$\{4, 10, 5, 11\}$
LL	$\{0, 6, 5, 11\}$
LP	$\{1, 7, 0, 6\}$
LPL	$\{0, 6\}$
PSMA	$\{1, 7, 2, 8\}$
PSTA	$\{12, 18, 1, 7, 2, 8\}$
MSTA	$\{2, 8\}$
AS	$\{2, 8, 3, 9\}$
RA	$\{3, 9, 4, 10, 16, 22, 17, 23\}$
RP	$\{12, 18\}$
RPL	$\{12, 18, 13, 19\}$
RL	$\{13, 19, 14, 20\}$
RAL	$\{14, 20, 15, 21, 16, 22\}$

Table S3. Map between our 24 anatomical regions and the 10 regions used by the DT algorithm presented by Arruda and coauthors in Arruda et al. (1998).

3 ROBUSTNESS OF LEAD IMPORTANCE TO LEAD-ORDER PERMUTATION AND RATIONALE FOR CIRCULAR PADDING

To verify that the resulting lead importance ranking given by (Eq. 5) reflects genuine lead-specific relevance rather than an artifact of the lead arrangement, i.e. column order, in the ECG image, we investigated robustness of lead importance to lead-permutation. Eleven lead orderings were considered: the original clinical ordering (I, II, III, aVR, aVL, aVF, V1, V2, V3, V4, V5, V6) and ten additional random permutations of the twelve leads as columns of the image. For each ordering, the 2D-FCN on which we propose to apply eXplainable Artificial Intelligence (XAI) methods was retrained from scratch, and the Guided Grad-CAM lead-importance ranking (rank 0 = most important, rank 11 = least important) was recomputed on the corresponding test set.

Other than varying the lead ordering, we introduced another axis along which trained models differed, namely the kernel size and padding in convolutions. For all lead permutations, models were trained under three paradigms: (i) square kernels and zero padding on the ECG samples; (ii) rectangular kernels spanning multiple time instants at a time but only one lead and (iii) square kernels and non-zero padding along the lead dimension in the ECG samples, so that no lead would be convolved with the null signal. Notice that contrary to (ii), in (i) and (iii) multiple leads enter the convolution at the same time, thus allowing the network to learn spatial relationships together with temporal ones. The rationale for going beyond (i) and

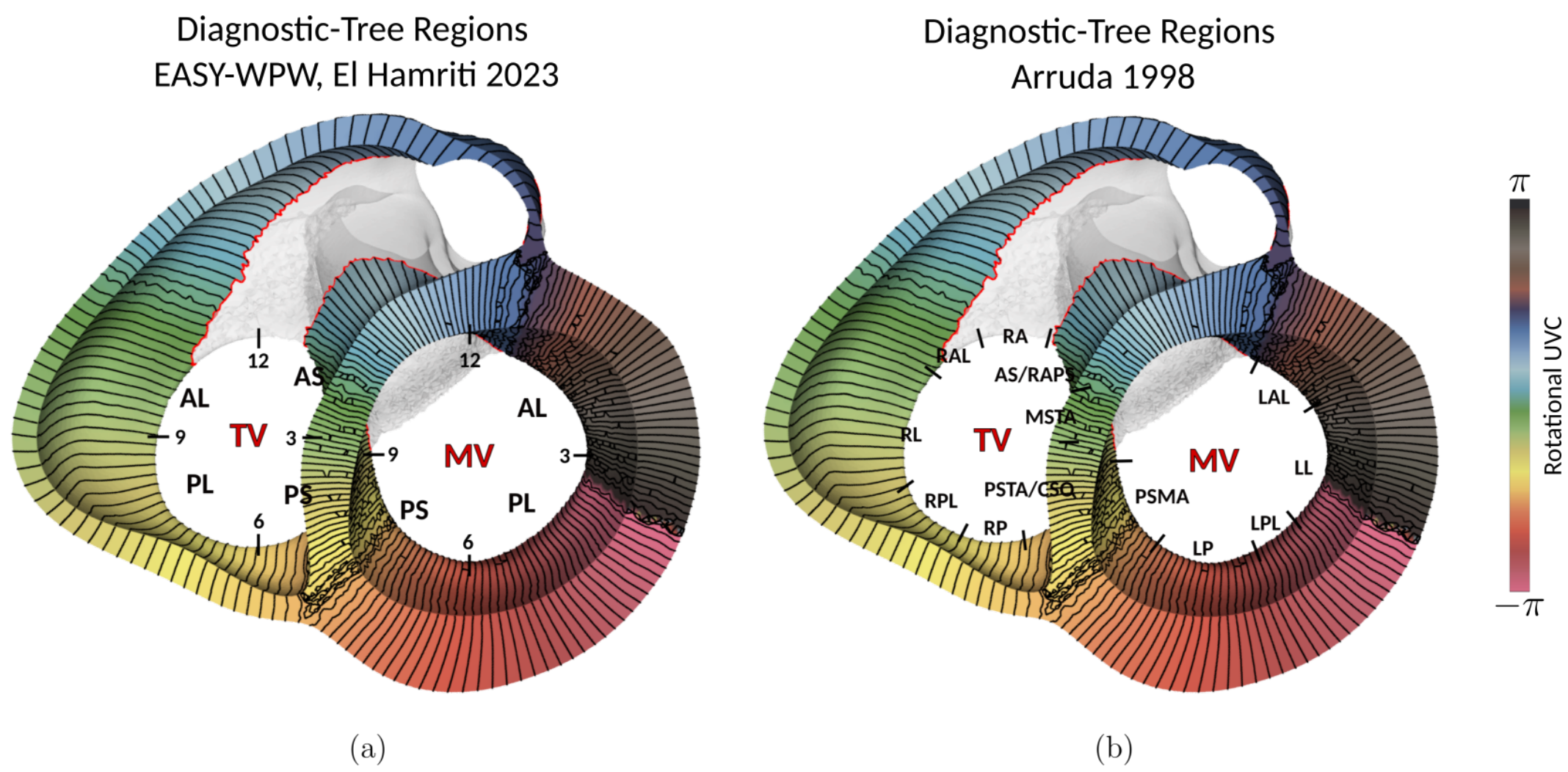

Figure S1. DT region subdivision according to EASY-WPW El Hamriti et al. (2023) algorithm (a) and Arruda Arruda et al. (1998) algorithm (b) on our ventricular model represented in a $60\,\mathrm{deg}$ left anterior oblique view. The colors represent the distribution of the rotational UVC.

introducing cases (ii) and (iii) follows from the observation that, under paradigm (i), the lead-importance ranking was not stable across the eleven permutations, as illustrated in the following. Indeed, a systematic quadratic relationship emerged between a lead's importance rank and its column position in the ECG image (Figure S2), with Grad-CAM, and to a lesser extent Guided Backpropagation, consistently favoring leads placed centrally in the image regardless of which physical lead occupied that position. This trend is a direct consequence of zero padding along the lead axis: leads placed at the leftmost and rightmost columns of the image are convolved with a receptive field that is partly filled with uninformative zero signals, whereas centrally-placed leads are not, an effect that compounds with the stacking of convolutional layers. Paradigms (ii) and (iii) were therefore introduced to remove this positional artifact from genuine lead importance. Paradigm (ii) avoids the artifact by construction, since single-lead rectangular kernels never mix information across leads and require no padding along the lead dimension. Paradigm (iii) instead retains the joint spatio-temporal encoding of (i), while removing the zero-padding artifact by replacing it with circular padding along the lead axis, so that every lead is convolved with an equally populated and physically meaningful neighborhood. For details on 2D convolutions and the meaning of circular padding see Section 2.2.3 of the main text.

Under both cases (ii) and (iii), the quadratic rank-versus-column-position relationship observed under paradigm (i) was destroyed (Figures S3 and S4), confirming that the trend observed under zero padding is attributable to the padding scheme itself rather than to genuine lead importance. However, the single-lead rectangular kernels achieved a lower classification accuracy (87%) than the square-kernel modes in (i) and (iii), a degradation limiting its usefulness for investigating lead importance. Square kernels and circular padding, in contrast, preserved the classification accuracy attained under square kernels and zero-padding (94.12% against 95.03% on the test set), while avoiding the positional artifact. On this basis, we adopted circular padding, rather than zero padding, for the 2D-FCN described throughout the manuscript. Finally, comparing results across permutations we observed that temporal localization of lead features is robust to lead ordering (see Figure S5).

Analyzing Guided Grad-CAM rankings obtained under paradigm (iii) across all 11 permutations, restricted to correctly classified test ECGs and separated by ventricle:

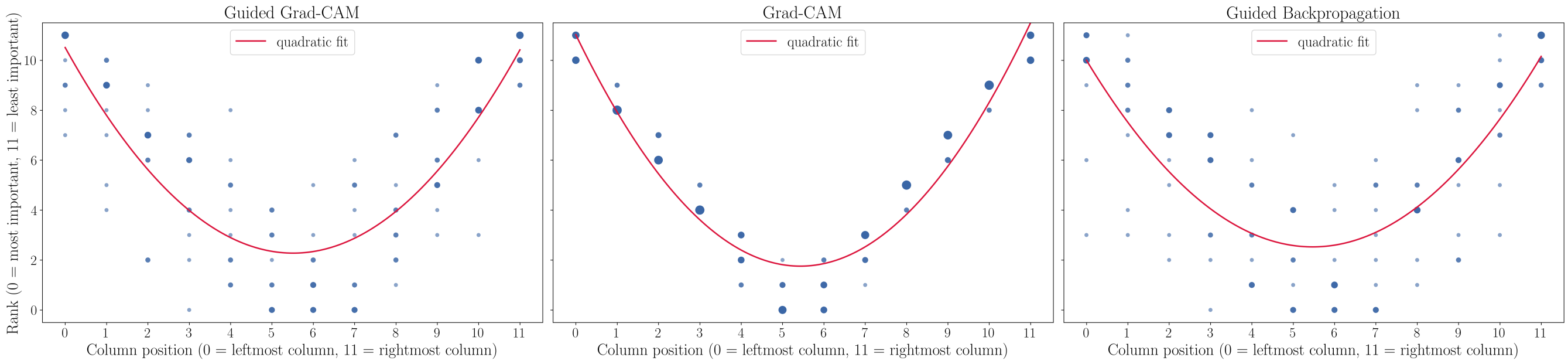

Figure S2. Importance rank versus column position in the ECG image under paradigm (i) (square kernels, zero padding). Borders of the image correspond to columns 0 and 11. Leads with a central placement are further from columns 0 and 11 and are consistently ranked more important (values closer to 0). The size of dots indicates frequency of assignment to a given rank by the lead importance metric.

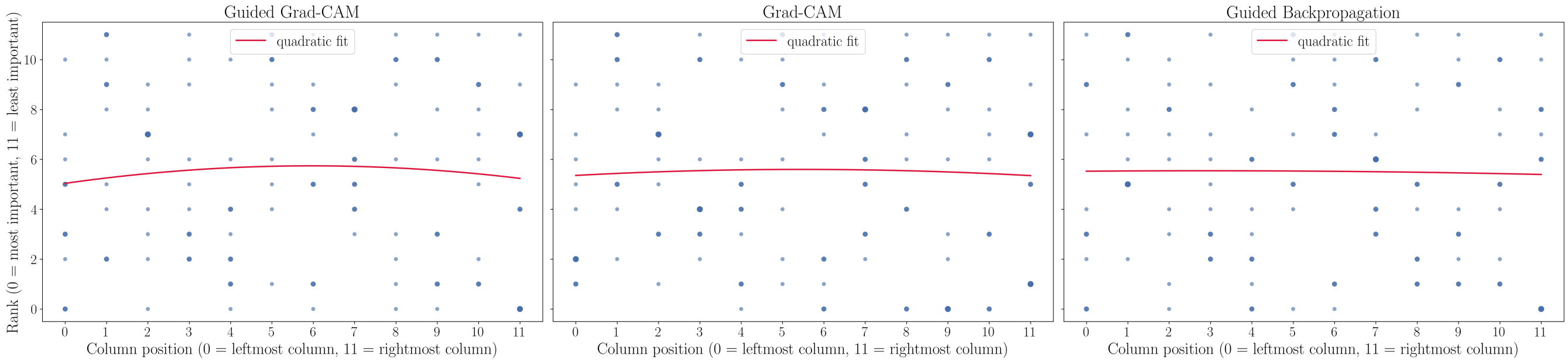

Figure S3. Importance rank versus column position in the ECG image under paradigm (ii) (rectangular, single-lead kernels). Borders of the image correspond to columns 0 and 11. The size of dots indicates frequency of assignment to a given rank by the lead importance metric. No lead is ranked more important than others just according to its position in the ECG image.

- left ventricle (LV): V2 is the most important lead in 8 out of the 11 permutations (rank 0), with aVR and aVL consistently ranked immediately after (ranks 1–2 in the majority of permutations); III is the next most important, while I, II, and V3 are consistently among the least important leads.
- right ventricle (RV): the ranking is markedly more stable, with V2 ranked most important (rank 0) in 10 out of 11 permutations and V1 ranked second (rank 1) in 10 out of 11 permutations; V5 is consistently the least important lead (rank 11 in 9 out of 11 permutations).

These results are consistent with, and support the robustness of, the lead-importance ranking reported in Section 3.3 of the main text. The complete ranking-frequency heatmaps across the three paradigms are reported in Figure S6.

4 CASCADING MODEL-RANDOMIZATION SANITY CHECK FOR SALIENCY MAPS

To assess whether the saliency maps depend on the learned model parameters, we performed the cascading model-randomization sanity check proposed in Adebayo et al. (2018) on the fully trained 2D-FCN model.

Starting from the fully trained network, layers were progressively reinitialized from the output towards the input. Specifically, the dense classification layer was first reinitialized, followed by the three convolutional blocks from the deepest to the shallowest. After each randomization step, saliency maps were recomputed

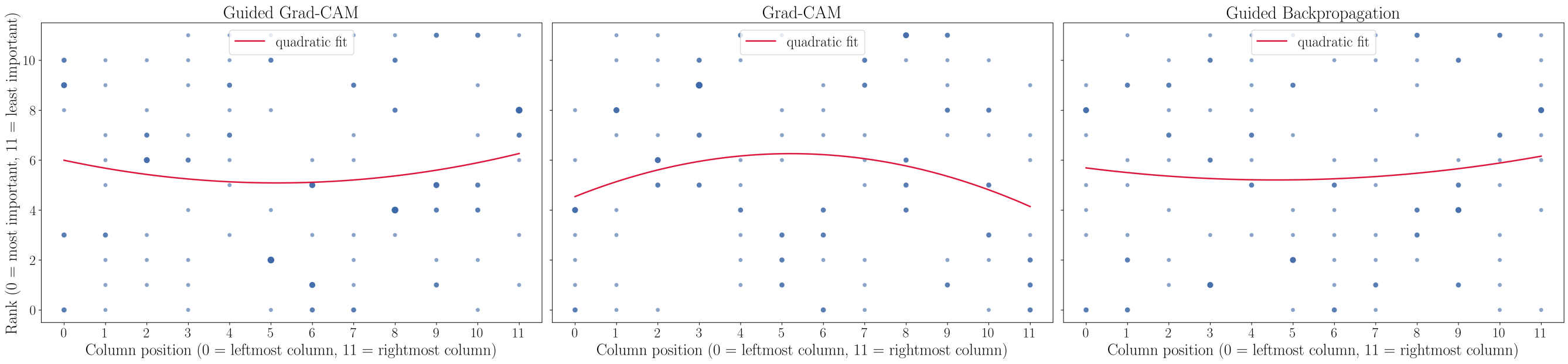

Figure S4. Importance rank versus column position in the ECG image under paradigm (iii) (square kernels, circular padding). Borders of the image correspond to columns 0 and 11. No systematic relationship between rank and column position remains.

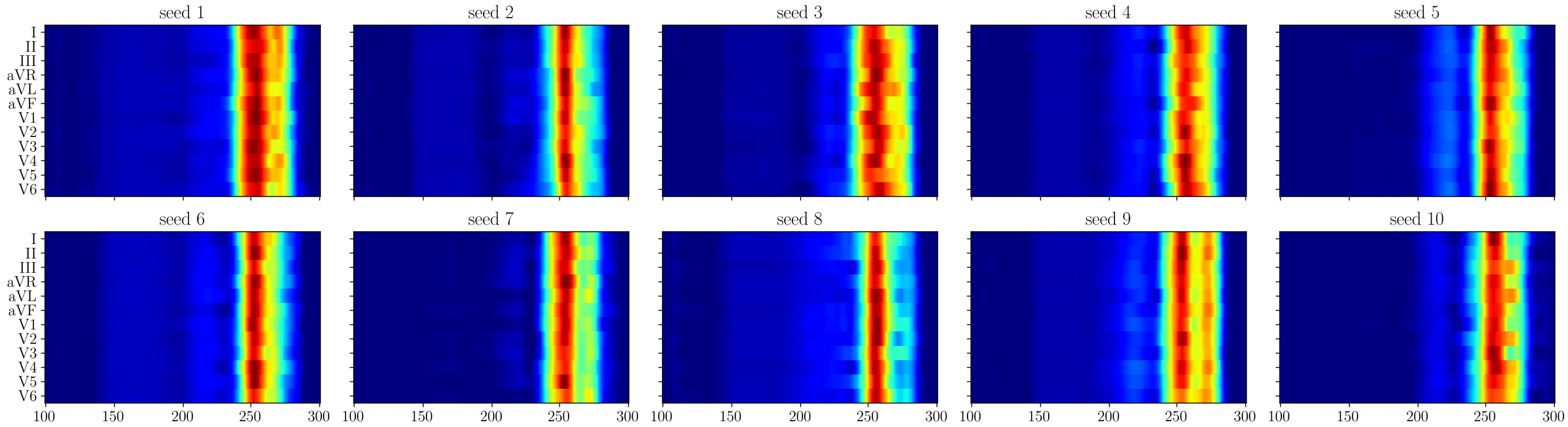

Figure S5. Grad-CAM saliency map for one test ECG, resulting from training on different lead permutations. For the sake of visualization all leads are reordered to follow one common arrangement. The map is visualized as an image with times along the width dimension. The important features are coarsely localized, as observed in computer vision literature. Across lead permutations, the temporal localization of important features is stable.

for the predicted class of 100 test ECGs and compared with those obtained from the original fully trained model using Spearman rank correlation ρ_s.

The analysis was performed separately for Grad-CAM, Guided Backpropagation, and Guided Grad-CAM. Consistent with previous observations in the computer vision literature, Grad-CAM passed the sanity check, with activation maps exhibiting progressively decreasing similarity to those of the fully trained model as layers were randomized. After complete network randomization, the saliency maps were only weakly correlated with those of the fully trained model ($\rho_s = 0.32$). In contrast, Guided Backpropagation degraded much more weakly, remaining substantially correlated with the original saliency maps even after complete parameter randomization ($\rho_s = 0.83$). This behavior is aligned with previous works describing Guided Backpropagation as acting primarily as an edge detector for image data. Despite the Spearman rank correlation being the main metric used in Adebayo et al. (2018), we remark that in our case, where most values in saliency maps are zero, high correlations are dominated by these zeros, hence large values would still be obtained under maps for which important regions differ, but the vast majority of zeros is found at the same pixel positions. This concept is illustrated in a synthetic example in Figure S7, where two simplistic saliency maps where the important features are located in completely different parts of the image are still largely correlated ($\rho_S = 0.94$), as the correlation is dominated by the unimportant regions

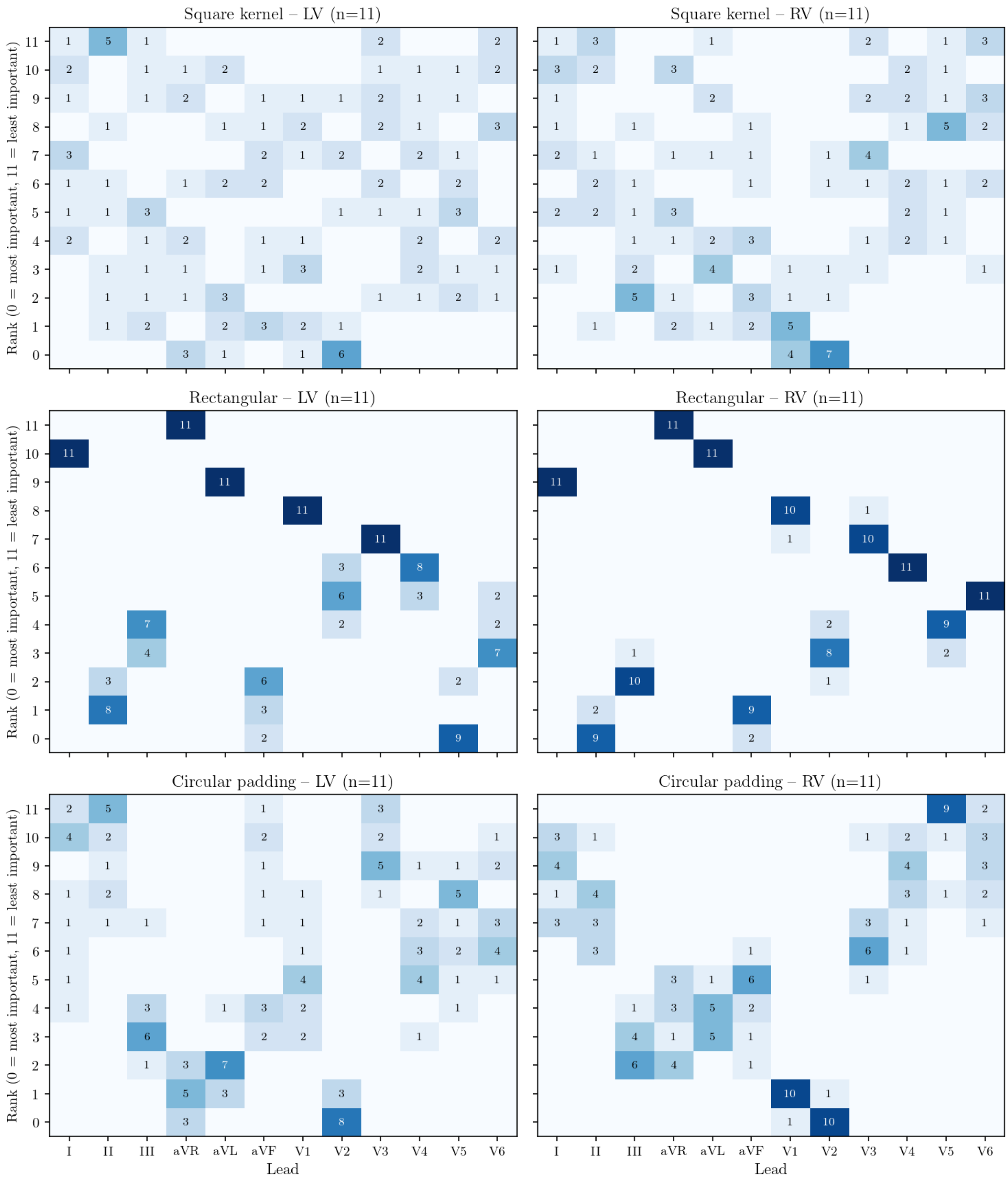

Figure S6. Ranking of leads according to lead importance across the 11 lead permutations. The heatmap reports the frequency with which a lead was assigned to each importance rank (rank 0 = most important, rank 11 = least important), for paradigms (i), (ii), and (iii).

overlapping almost everywhere. Therefore, we also inspect visually degraded saliency maps to confirm the described degradation behavior. Examples are reported in Figure S8.

The primary explainability method adopted throughout this work was Guided Grad-CAM, rather than Grad-CAM or Guided Backpropagation alone. The saliency maps yielded by Guided Grad-CAM also exhibited degradation under cascading randomization, reaching a Spearman correlation of $\rho_s = 0.27$

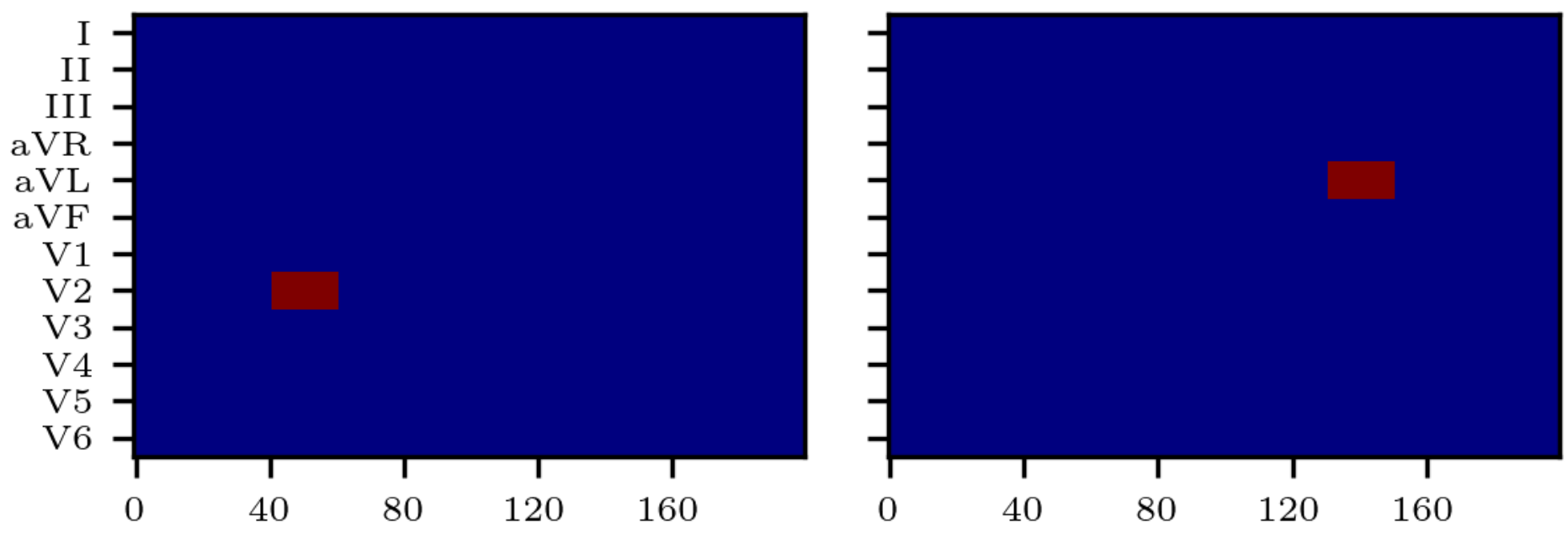

Figure S7. Two exemplary synthetic saliency maps whose Spearman rank correlation is large despite disagreement on important pixels.

with the saliency maps produced by the fully trained model after complete network randomization. This indicates that its outputs remain sensitive to the learned model parameters. We therefore attribute the parameter sensitivity of Guided Grad-CAM to its Grad-CAM component, while exploiting Guided Backpropagation as a refinement to obtain sharper visualizations. This design choice was motivated by the complementary characteristics of the two methods. Grad-CAM alone produced broad and diffuse activation maps (Figure S5), making it difficult to precisely localize in time and across leads the waveform components driving the prediction. Although Guided Backpropagation by itself did not satisfy the sanity check to the same extent as Grad-CAM, it was retained within Guided Grad-CAM to refine the visual localization of potentially physiologically meaningful regions. This refinement was considered important because the resulting explanations were subsequently assessed by a cardiologist to determine the clinical plausibility of the highlighted ECG features.

Finally, expert assessment indicated that the highlighted waveform regions were physiologically meaningful. For this reason, we retained Guided Grad-CAM as the primary XAI method throughout this work: the Grad-CAM component provides sensitivity to the learned model parameters, while the Guided Backpropagation component enhances the visual localization required for expert interpretation.

REFERENCES

El Hamriti M, Braun M, Molatta S, Imnadze G, Khalaph M, Lucas P, et al. EASY-WPW: A novel ECG-algorithm for easy and reliable localization of manifest accessory pathways in children and adults. *Europace: European Pacing, Arrhythmias, and Cardiac Electrophysiology: Journal of the Working Groups on Cardiac Pacing, Arrhythmias, and Cardiac Cellular Electrophysiology of the European Society of Cardiology* **25** (2023) 600–609. doi:10.1093/europace/euac216.

Arruda MS, McClelland JH, Wang X, Beckman KJ, Widman LE, Gonzalez MD, et al. Development and validation of an ECG algorithm for identifying accessory pathway ablation site in Wolff-Parkinson-White syndrome. *Journal of Cardiovascular Electrophysiology* **9** (1998) 2–12. doi:10.1111/j.1540-8167.1998.tb00861.x.

Adebayo J, Gilmer J, Muelly M, Goodfellow I, Hardt M, Kim B. Sanity checks for saliency maps. *Advances in neural information processing systems* **31** (2018).

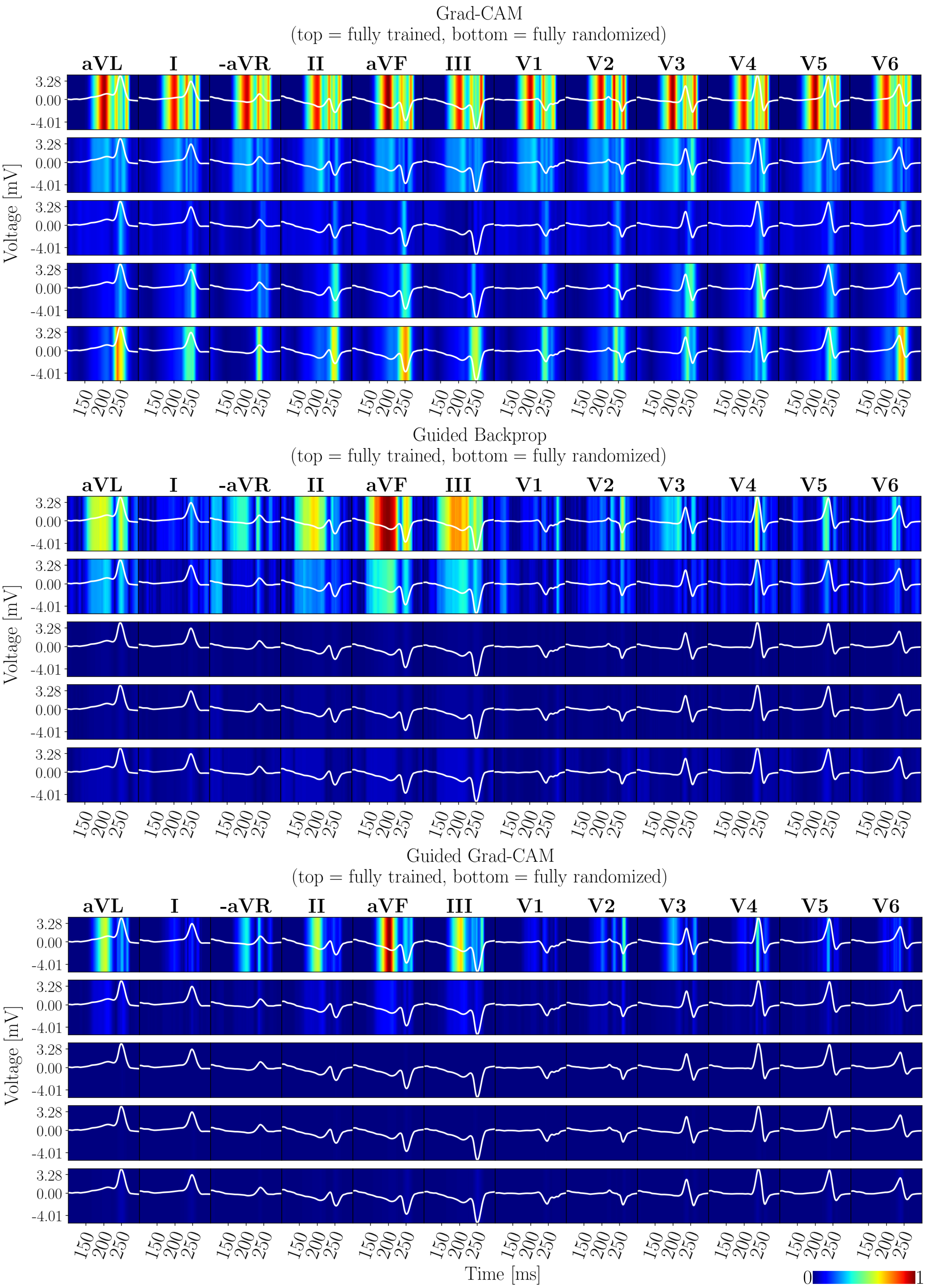

Figure S8. Example of saliency maps variability with cascading randomization of weights.
